# *MAGEB2* is Activated by Promoter Demethylation in Head and Neck Squamous Cell Carcinoma

**DOI:** 10.1371/journal.pone.0045534

**Published:** 2012-09-24

**Authors:** Kavita M. Pattani, Ethan Soudry, Chad A. Glazer, Michael F. Ochs, Hao Wang, Juliana Schussel, Wenyue Sun, Patrick Hennessey, Wojciech Mydlarz, Myriam Loyo, Semra Demokan, Ian M. Smith, Joseph A. Califano

**Affiliations:** 1 Department of Otolaryngology—Head and Neck Surgery, Johns Hopkins Medical Institutions, Baltimore, Maryland, United States of America; 2 Department of Basic Oncology, Oncology Institute, Istanbul University, Capa, Istanbul, Turkey; 3 Department of Oncology Biostatistics, Johns Hopkins Medical Institutions, Baltimore, Maryland, United States of America; 4 Milton J. Dance Head and Neck Center, Greater Baltimore Medical Center, Baltimore, Maryland, United States of America; The Chinese University of Hong Kong, Hong Kong

## Abstract

**Purpose:**

Although promoter hypermethylation has been an accepted means of tumor suppressor gene inactivation, activation of otherwise normally repressed proto-oncogenes by promoter demethylation has been infrequently documented.

**Experimental Design:**

In this study we performed an integrative, whole-genome analysis for discovery of epigenetically activated proto-oncogenes in head and neck cancer tumors. We used the 47K GeneChip U133 Plus 2.0 Affymetrix expression microarray platform to obtain re-expression data from 5-aza treated normal cell line and expression data from primary head and neck squamous cell carcinoma (HNSCC) tumor tissues and normal mucosa tissues. We then investigated candidate genes by screening promoter regions for CpG islands and bisulfite sequencing followed by QUMSP and RT PCR for the best candidate genes. Finally, functional studies were performed on the top candidate gene.

**Results:**

From the top 178 screened candidates 96 had CpG islands in their promoter region. Seven candidate genes showed promoter region methylation in normal mucosa samples and promoter demethylation in a small cohort of primary HNSCC tissues. We then studied the demethylation of the top 3 candidate genes in an expanded cohort of 76 HNSCC tissue samples and 17 normal mucosa samples. We identified *MAGEB2* as having significant promoter demethylation in primary head and neck squamous cell carcinoma tissues. We then found significantly higher expression of *MAGEB2* in tumors in a separate cohort of 73 primary HNSCC tissues and 31 normal tissues. Finally, we found that *MAGEB2* has growth promoting effects on minimally transformed oral keratinocyte cell lines but not a definite effect on HNSCC cell lines.

**Conclusion:**

In conclusion, we identified *MAGEB2* as activated by promoter demethylation in HNSCCand demonstrates growth promoting effects in a minimally transformed oral keratinocyte cell line. More studies are needed to evaluate *MAGBE2's* exact role in HNSCC.

## Introduction

Epigenetic modifications encompass changes such as DNA methylation, chromatin modifications, and genomic imprinting. Aberrant promoter hypermethylation has been well-characterized by comprehensive whole-genome profiling approaches to identify novel tumor suppressor genes (TSGs) that are silenced in this manner. This loss of gene function can provide a selective advantage to cancer cells. On the other hand, promoter demethylation resulting in activation of proto-oncogenes has only been sporadically reported [1], [2], [3], [4]. More recently, we have employed pharmacologic re-expression techniques coupled with primary tumor expression analysis [2], [3] and have demonstrated coordinated promoter demthylation and reactivation of epigenetically silenced genes in head and neck cancers and lung cancers.

Head and neck cancer accounts for 3–5% of all new cancer cases in the United States. The American Cancer Society estimates the incidence of head and neck cancer to be 49,260 new cases with an estimated 11,480 deaths in 2010 [5]. Significant research is currently ongoing in uncovering the molecular mechanisms and further characterizing the role of epigenetic changes in the pathogenesis of development of head and neck squamous cell carcinomas (HNSCC).

**Figure 1:**
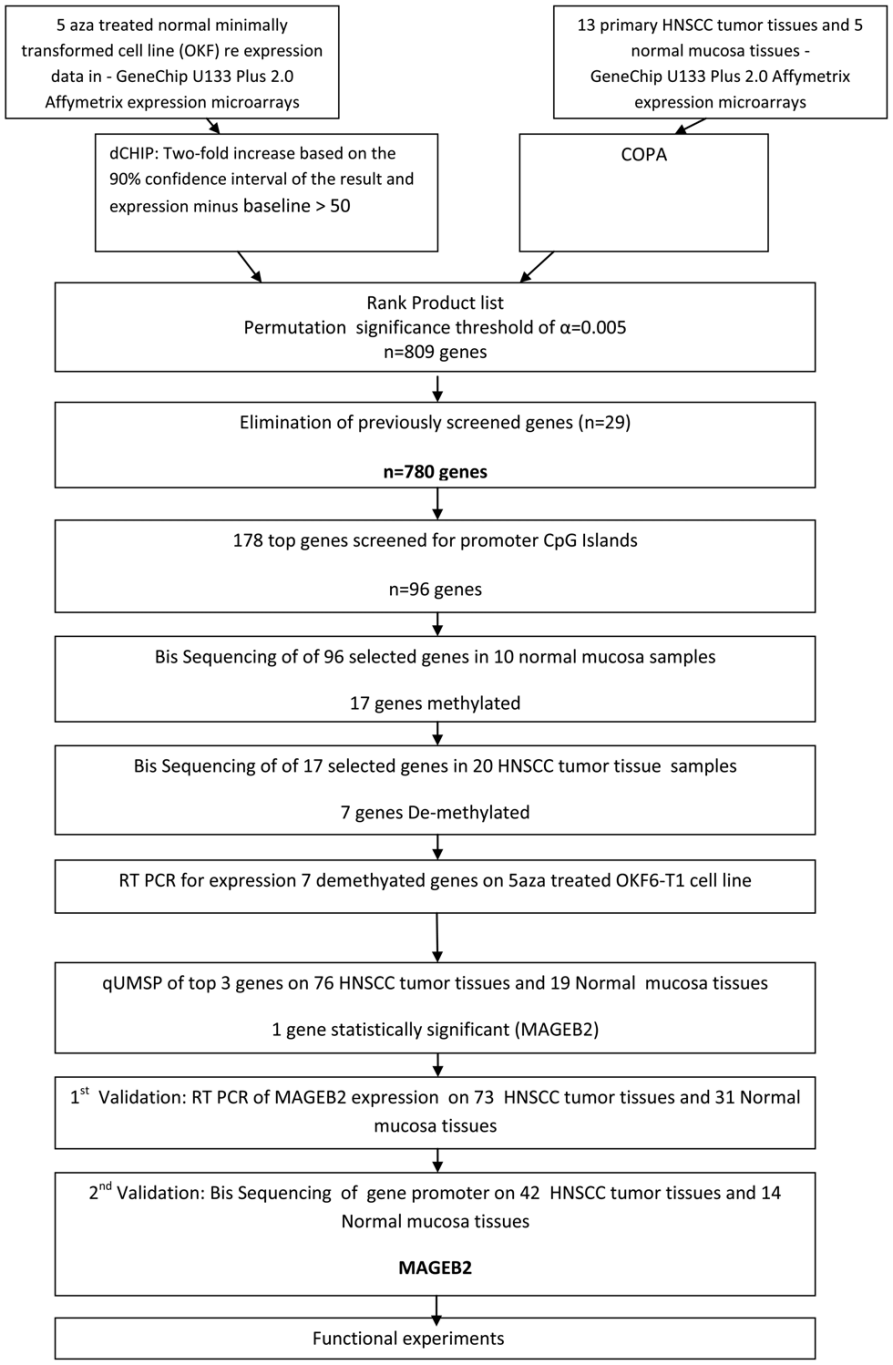
Study Flowchart.

Our lab has previously demonstrated activation of proto-oncogenes via promoter demethylation in a small cohort of head and neck tumor samples. In this study, we expanded our efforts in identifying targets that were activated by promoter demethylation and upregulated in a larger cohort of HNSCC and aza treated normal oral keratinocyte cell line, employing a larger 47K expression array platform to obtain expanded gene coverage for discovery (Figure 1).

## Materials and Methods

### Patient Samples

Tumor and normal tissue samples were collected from HNSCC patients and from healthy non cancer patients at Johns Hopkins Hospital. Tissues were obtained via Johns Hopkins Institutional Review Board approved protocols. Written informed consent was obtained from each subject prior to the tissue harvesting and use for scientific research. All samples were analyzed by the pathology department at Johns Hopkins Hospital. Normal samples were microdissected and DNA prepared from the normal mucosa from the head and neck. Tumor samples were confirmed to be HNSCC and subsequently microdissected to at least 75% purity and genomic DNA was extracted as described below.

#### 5Aza-dC and TSA Treatment of Cells

These *in vitro* techniques employ treatment of cultured cells with 5-aza-deoxycytidine (a cytosine analog which cannot be methylated) with or without Trichostatin A (a histone deacetylase inhibitor) and subsequent expression array analysis with validation of tumor suppressor gene targets. We treated normal oral kertinocyte cell lines (OKF6-Tert1, immortalized with hTert, a generous gift from J. Rheinwald, Harvard) with 5Aza-dC and TSA in duplicate as described previously [6]. Briefly, cells were split to low density (1×10^6^ cells/T-75 flask) 24 hours before treatment. Stock solutions of 5Aza-dC (Sigma, St. Louis, MO) and TSA (Sigma) were dissolved in 50% acetic acid and 100% ethanol, respectively. Cells were treated with 5 ul of 5 uM 5-Aza-deoxycytidine for 4 days and 3 ul of 300 nM TSA was added for the last 24 hours. Baseline expression was established by mock-treated cells with the same volume of 50% acetic acid or ethanol.

#### RNA Extraction and Oligonucleotide Microarray Analysis

Total cellular RNA was isolated using Trizol (Life Technologies, Gaithersburg, MD) and the RNeasy kit (Qiagen, Valencia, CA) according to the manufacturer's instructions. We carried out oligonucleotide microarray analysis using the GeneChip U133 Plus 2.0 Affymetrix expression microarray (Affymetrix, Santa Clara, CA) on our AZA/TSA and mock treated normal cell lines and on a separate cohort of 13 HNSCC primary tumor tissues and 5 normal mucosa tissues. The U133A Plus 2.0 microarray platform (Affymetrix, Santa Clara California) covers over 47,000 transcripts. Samples were converted to labeled, fragmented cRNA per the Affymetrix protocol for use on the expression microarray.

For the cell lines arrays data, signal intensity and statistical significance was established for each transcript using dChip version 2005. Two-fold increase based on the 90% confidence interval of the result and expression minus baseline >50 was used as the statistical cutoff value to identify upregulated candidate genes. For the tissue arrays we applied the Cancer Outlier Profile Analysis method.

#### Cancer Outlier Profile Analysis (COPA)

We applied COPA to our cohort of 18 tissues (13 tumors, 5 normals) (Figure S1). For details of the method refer to Tomlins et. al. [7]. Briefly, gene expression values were median centered, setting each gene's median expression value to zero. The median absolute deviation (MAD) was calculated and scaled to 1 by dividing each gene expression value by its MAD. Of note, median and MAD were used for transformation as opposed to mean and standard deviation so that outlier expression values do not unduly influence the distribution estimates, and are thus preserved post-normalization. Finally, the 75th, 90th, and 95th percentiles of the transformed expression values were calculated for each gene, and then genes were rank-ordered by their percentile scores, providing a prioritized list of outlier profiles. For the purposes of our rank-list, the 90th percentile was chosen based on sample-size analysis.

#### Integrative Analysis

We created one rank list based on COPA upregulation at the 90th percentile of the target genes from the Affymetrix U133A Plus 2.0 mRNA expression microarray platform used on the cohort of 13 primary HNSCC tissues and 5 normal tissues. A second rank list was produced by ranking genes in descending order of the degree of upfold regulation upon 5-aza/TSA treatment of OKF6-Tert1 cell line. These two rankings were then combined using a rank product (rank 1×rank 2) to rank all targets and permutation of the data was used to establish significance with a threshold of α = 0.005. The lower the rank product score the higher the gene was ranked. The best performing of these targets were comprehensively evaluated first by obtaining the genomic sequences from UCSC genome browser. Next, the presence of CpG islands in these genes was determined by MethPrimer which relies on Island size >100, GC Percent >50.0%, Obs/Exp >0.60. The information from these target genes were then crossed with the previously screened genes by our lab [3] in order to eliminate overlapping genes to give us a final ranked list of candidate genes.

#### DNA Extraction

Samples were digested with 1% SDS and 50 µg/mL proteinase K (Boehringer Mannheim) at 48°C overnight, followed by phenol/chloroform extraction and ethanol precipitation of DNA as previously described.

#### Bisulfite Treatment

DNA was subjected to bisulfite treatment. Briefly, 1–2 µg of genomic DNA from HNSCC tissues and normal mucosa tissues were subjected to bisulfite treatment using the EpiTect® Bisulfite Kit (Qiagen, Valencia, CA) according to the manufacturer's instructions. This bisulfite treated DNA was then stored at −80°C.

#### Bisulfite Sequencing

Bisulfite sequence analysis was performed to verify the methylation status in primary HNSCC tumors and non smoking normal mucosa tissues (UPPP). Bisulfite-treated DNA was amplified using bisulfite sequencing primers designed by MethPrimer to span areas of CpG islands in the promoter or first exon [8]. Primer sequences were designed to not contain CG dinucleotides. Detailed primer sequences and PCR conditions are available upon request. The PCR products were gel-purified using the QIAquick 96 PCR Purification Kit (Qiagen, Valencia, CA), according to the manufacturer's instructions. The purified PCR products were sequenced with both forward and reverse primers by GENEWIZ™.

#### Quantitative RT-PCR (QRT-PCR)

1 ug of RNA was then utilized for cDNA synthesis. Reverse transcription was carried out using SuperScript First- Strand Synthesis kit (Invitrogen) and cDNA High Capacity Kit (Applied Biosystems). The final cDNA products were used as the templates for subsequent RT-PCR.

RT PCR was performed either with SYBR Green technology (Quantifast SYBR Green PCR Kit (Qiagen, Valencia, CA)) using primer sets (Table S1) that were designed using Primer3 and Integrated DNA techniques (idtdna.com) or with readymade primers-probe mix from Applied Biosystems (Assay Hs00427156_m1, Assay Hs03928990_g1). 18 s rRNA was examined to ensure accurate relative quantification in qRT-PCR. Serial dilutions of cell line cDNA, showing to express the specific gene studied, were used as positive controls to create standard curves. Each experiment was performed in triplicate using the ABI 7900HT real-time PCR machine. Expression values are presented as gene of interest (GOI)/18SX100.

#### Quantitative Unmethylation Specific PCR (QUMSP)

To selectively amplify demethylated promoter regions in genes of interest, probe and primers were designed using data from bisulfite sequencing of primary tumors which are complimentary only to bisulfite-converted sequences known to be demethylated in tumor. Primer-probe sets were designed specifically for each candidate gene using MethPrimer and Integrated DNA techniques (idtdna.com). Probe and primer combinations were validated using *in vitro* methylated and demethylated controls. All experiments were performed in triplicate using the ABI 7900HT real-time PCR machine with standard curves normalized to *BACTIN* primers that do not contain CpG islands in the sequence for quantification to relative DNA input. Unmethylation values are presented as % unmethylation [GOI/BACTIN X100]. Probe and primer sequences are listed in Table S2.

#### Transfection of human expression vectors and anchorage dependent growth assay

A full-length ORF cDNA plasmids of *MAGEAB2* (SKU: SC122625) was obtained for transient transfections from OriGene (Rockville, MD). The amplified cDNA was purified using the Midiprep Kit (Qiagen, Valencia, CA). Cell lines- NOKSI (normal oral keratynocyte spontaneously immortalized) and O22, 011, 028 and Fadu(HNSCC cell lines) were plated at 1.5×10^5^/well using 6-well plates and transfected with either empty vector or gene of interest using the FuGene 6 Transfection Reagent (Roche, Basel, Switzerland) according to the manufacturer's instructions. Cell Counting Kit-8 (CCK-8) (Dojindo, Rockville, MD) absorbance was then measured by the Spectramax M2e 96-well fluorescence plate reader Molecular Devices (Sunnyvale, CA). All anchorage dependent growth experiments were performed in triplicate. Growth curves were measured at 0, 24, 48 and 72 hours using CCK-8 absorbance and subsequent RT-PCR reactions (normalized to 18 s RNA) were performed to reveal overexpression.

### Colony Formation Assay

Forty-eight hours after transfection with wild-type, mutant, or control constructs, cells (1×105) were seeded into 100-mm Petri dishes with 10 ml of RPMI supplemented with 10% FBS and 500 µg/ml G418. After 14 days, the resulting colonies were rinsed with PBS, fixed with methanol, and stained with Giemsa (Sigma, St. Louis, MO). The number of colonies per dish was counted. All experiments were performed in triplicate, and standard deviations were calculated.

#### Soft-Agar Assay

Soft-agar assays were performed on six-well plates. Forty-eight hours after transfection with wild-type, mutant, or control constructs, cells (5×103) were mixed with 1 ml of RPMI with 0.3% low-melting agarose and 10% FBS supplemented with 500 µg/ml G418 and poured onto a bed of 1 ml per well DMEM with 0.5% agarose and 10% FBS supplemented with 1,000 µg/ml G418. After 18 days, colonies were counted with the Nikon SMZ1500 microscope and photographed with the Nikon DXM camera. All experiments were performed in triplicate, and standard deviations were calculated.

### Statistical Analysis

The QUMSP and RT-PCR data were analyzed using the non parametric Wilcoxon test. For bisulfite sequencing data, a summarized methylation score was computed as the percentage of methylated CpGs among the total CpGs examined. The comparison of tumor and normal samples in terms of the methylation score was done using the Spearmen rank correlation was used to analyze the association between percentage of methylated CpGs and expression. Tests with p values<0.05 were considered significant. The analysis was performed using SAS®, version 9.1 (Cary, NC).

As a further test and to correct for missing data, we estimated methylation status for cases where we could not measure the methylation by assigning a methylation value equal to the probability that the site was methylated based on all measured samples. We then summed across all 18 CpG sites to obtain a numerical value for the methylation, ranging between 3 and 18, for each sample. We binarized the methylation data around 10 sites and the expression data as greater or less than 1, and used a Fisher Exact Test on the 2×2 contingency table that resulted to estimate significance.

## Results

### Integrative Discovery Approach of Activated Proto-oncogenes

Using an integrative high-throughput discovery approach, previously described [2], [3] we screened for activated proto-oncogenes in HNSCC tissues using: 1) re expression data of 5-aza treated normal minimally transformed cell line (OKF) in a GeneChip U133 Plus 2.0 Affymetrix expression microarray platform and 2) expression data from 13 primary HNSCC tumor tissues and 5 normal mucosa tissues performed on the same platform.

These two sources of information (gene set demonstrating upregulation with 5-aza and COPA score for the tissue microarrays) were then combined by calculating a rank product (the lower the rank product score the higher the gene was ranked), and permutation of the data was used to establish significance with a threshold of alpha = 0.005. This resulted in 809 genes deemed significant (Table S3). Although a less stringent threshold may still have yielded significant gene candidates, we purposefully chose a stringent threshold to limit the complexity of subsequent analysis due to resource constraints. We then eliminated the 29 genes that were previously screened by our lab [3] resulting in a finally ranked list of candidate genes. The top 178 of these targets were comprehensively evaluated first by obtaining promoter (1000 bp upstream of TSS and first Exon) genomic sequences from UCSC genome browser (Table S4).

### Validation of Promoter Methylation of Genes in Normal Tissue Samples

We empirically screened the top 178 genes for presence of promoter CpG islands using UCSC Genome resulting in 96 genes that had promoter-associated CpG islands (table S5).

Utilizing MethPrimer we selected the appropriate primers for Bisulfite Sequencing (BSS). We performed BSS in 10 normal mucosa samples from patients undergoing uvulopalatopharyngoplasty without a diagnosis of cancer to confirm promoter constitutive baseline methylation in these tissues. Forward and reverse sequencing for all 96 genes in 10 mucosal samples were analyzed. 17 of the 96 candidate genes promoters were found to be consistently methylated (>75% of CpGs methylated) in *normal* tissue samples (Table 1).

**Table 1:** Detailed list of candidate gene promoters that were found to be consistently methylated (>75% of CpGs methylated) in *normal* tissue samples.

Accession	Symbol	Description	COPA Score	Methylated in Normal UPPP Mucosal Tissue	Unmethylated in HNSCC Tumor Tissue
NM_002364	*MAGEB2*	melanoma antigen family B,2	17	Y	Y
AL136861	CrispL	cysteine-rich secretory protein LCCL domain	19	Y	Y
X99142	KRT86	keratin hair basic 6 monilethrix	28	Y	N
W69083	KIPV467	KIPV467	38	Y	N
NM_002281	KRT81	keratin hair basic 1	48	Y	N
AF059274	CSPG5	chondroitin sulfate proteoglycan 5	55	Y	N
AA156998	PP1R14A	protein phosphatase 1 regulatory inhibitor 14	62	Y	Y
AI819198	KISS1R	G protein coupled receptor 54	64	Y	N
AK093300	KIAA1937	KIAA1937	85	Y	N
NM_007017	SOX30	SRY sex determining region Y-box 30	93	Y	Y
NM_018665	DEAD	Asp-Glu-Ala-Asp box polypeptide	119	Y	Y
NM_021083	KBGP	Kell Blood group precursor	121	Y	Y
AB076563	RLX3	relaxin 3	133	Y	N
AW269746	COX8C	cytochrome c oxidase subunit 8C	157	Y	Y
NM_014398	LAMP3	lysosomal-associated membrane protein 3	165	Y	N
AL136755	HORMAD1	HORMA domain containing 1	167	Y	N
U88667	ABCA4	ATP-binding-cassette sub-family A	169	Y	N

### Validation of Promoter *De*methylation of Genes in Tumors

**Figure 2:**
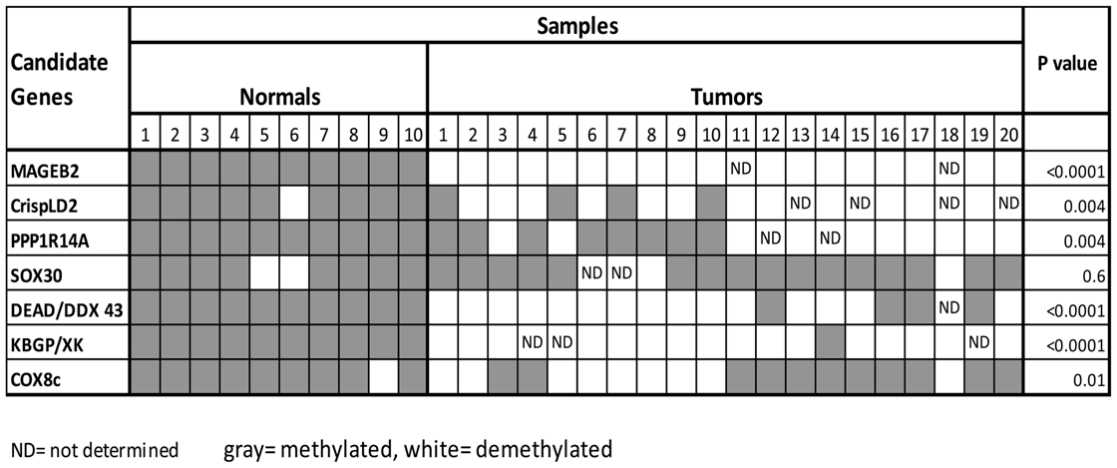
Promoter methylation status in preliminary cohort of 10 normal mucosa and 20 primary HNSCCS tissue samples. A sample was considered methylated when >75% of the sequenced CpGs were methylated. Spearmen rank correlation was used to analyze the association between percentage of methylated CpGs and expression. Gray = methylated, White = unmethylated, ND = undetermined.

Next we performed BSS using these 17 candidate genes in 20 tumor samples. Ten genes showed complete methylation in the tumor samples. Seven genes showed partial de-methylation in the tumor tissues including: *MAGEB2*, *CrispLD2*, *PPP1R14A*, *SOX30*, *DEAD*, *KBGP*, *COX8C*. Of these seven genes, all but *SOX30*, had shown to be statistically significant demethylated in tumors compared to normal mucosa samples (Figure 2).

### Validation of upfold expression of genes on OKF6-T1 cell line following 5-aza treatment

To validate the upfold re-expression of the above 7 genes that showed demethylation in tumor tissues we performed RT-PCR on 5-aza and mock treated OKF1 T cell line.

All genes showed upfold regulation, particularly *MAGEB2*, *DEAD/DDX43*, *KBGP/XK* and *PPP1R14A* (Figure S2).

### Qualitative UnMethylation Specific PCR Validation of Demethylated genes

**Figure 3:**
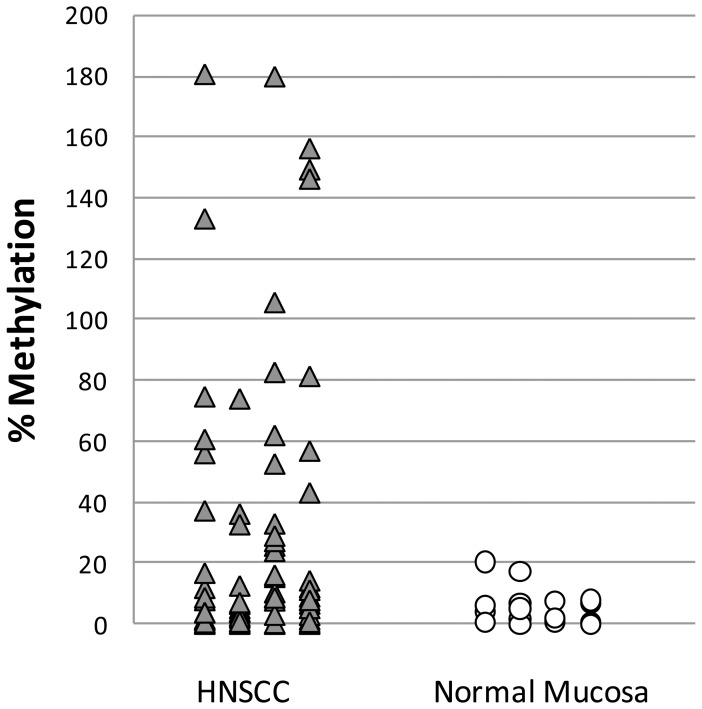
Scatter plot of QUMSP of MAGEB2.

In order to confirm the bisulfite sequencing results of our target genes and to provide a dataset of continuous variables to express the status of promoter demethylation, we performed QUMSP, which specifically measures non-methylated promoters. We assayed bisulfite extracted DNA from 76 HNSCC tumor tissues and 17 normal mucosa samples for *MAGEB2*, *KBGP/XK*, *DEAD/DDX43* which were found according to the preliminary BSS to be the most highly significant and showed high upfold expression values in AZA treated OKF6-T1 cell line (Figure 3, Table S6). Only for *MAGEB2* there was a trend of significant promoter demethylation in HNSCC tumor samples compared to normal mucosa samples (non parametric Wilcoxon test p = 0.08). P-values for *KBGP/XK and DEAD/DDX43* were 0.46 and 0.22 respectively.

### Assessment of gene expression in HNSCC tumor tissues Vs normal mucosa controls

**Figure 4:**
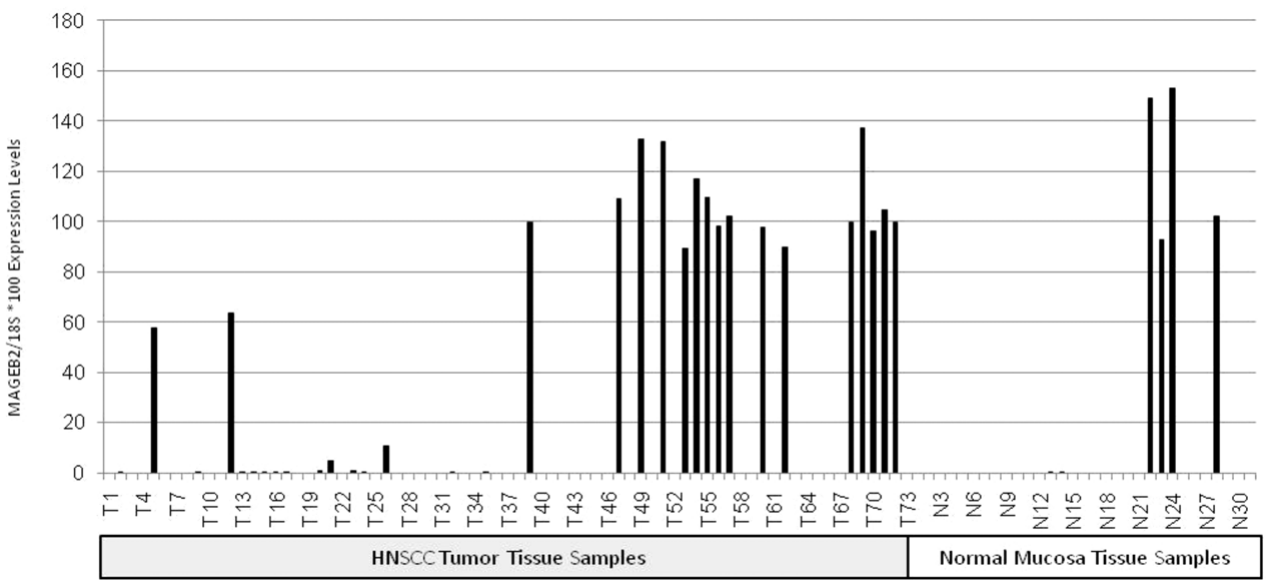
RTPCR *MAGEB2* expression in HNSCC and normal mucosa tissues.

Since only *MAGEB2* showed near statistical significance for promoter demethylation in tumors we proceeded to assess if promoter demethylation of *MAGEB2* is associated with tumor specific increased *MAGEB2* gene expression. We performed RT-PCR on a separate cohort of 73 HNSCC tumor tissues and 31 normal mucosa tissues (Figure 4, Table S7). Statistical analysis revealed significant overexpression of MAGEB2 in HNSCC (non parametric Wilcoxon test p = 0.037).

### Validation of the correlation between promoter demethylation and increased expression in HSNCC versus Normal controls

We next wanted to corroborate that promoter demethylation was responsible for re-expression of *MAGEB2*. We performed BSS of the CpG island in the promoter region on a subset of the previous cohort tested for *MAGEB2* expression (41 HNSCC tumor tissues and 13 normal mucosa tissues). All normal mucosa tissues showed complete methylation of the sequenced region and in all but two samples with extremely low gene expression, there was no detectable expression. In the tumor samples there were variable promoter demethylation and expression results (Figure S3). In order to correct for missing data, we estimated methylation at unmeasured CpGs as equal to the probability that the site was methylated based on all measured samples and summed the number of methylated sites for each sample. Given a dichotomy in the data we binarized methylation as methylated for values greater than 10 and unmethylated for values less than 10. Expression showed either expression significantly greater than 1 or near zero, so we performed binarization around this value for expression. Using a Fisher exact test, we obtained a p-value below 0.0006 with a 95 percent confidence interval of 0.00033–0.269.

### *MAGEB2* is Growth Promoting in Normal and HNSCC cell lines

**Figure 5:**
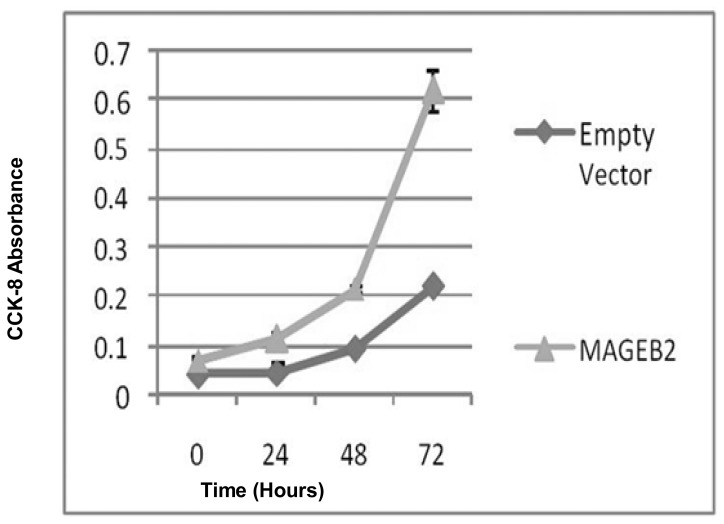
Anchorage dependent growth assays of *MAGEB2* in NOKSI cell line. NOKSI AD Growth at 72 hours post transient transfection with *MAGEB2*.

Anchorage Dependent Transient transfections were performed in NOKSI minimally transformed oral keratinocyte cell line and on O22, 011, 028 and Fadu HNSCC cell lines. Analysis of NOKSI growth at 72 h post transient transfection with *MAGEB2* revealed 179% (+/−36%) increase in growth (Figure 5). The expression data of *MAGEB2* in this experiment is shown in Figure S4. Anchorage dependent and independent transient transfections for the HNSCC cell lines tested did not, however, reveal growth promoting effects (Data not presented).

## Discussion

Promoter CpG island hypermethylation and silencing of tumor suppressor genes has been studied extensively in HNSCC [9], [10], [11], [12], [13], [14], [15], [16], [17], [18], [19], [20], [21], [22]. In contrast, only a limited number of studies examined the role of promoter *de*methylation and activation of proto-oncogenes in HNSCC and other malignancies. In previous reports from our lab [2], [3] we discovered activation of novel putative proto-oncogenes via promoter demethylation in HNSCC and lung cancer, particularly the *MAGE-A* gene family members.

In this study we extended our efforts in identifying targets that were activated by promoter demethylation and upregulated in HNSCC. We carried out oligonucleotide microarrays using the more comprehensive GeneChip U133 plus 2.0 Affymetrix expression microarray platform which covers over 47,000 transcripts on 1) primary HNSCC tumor tissues and normal mucosa tissues and on 2) normal oral keratinocyte cell lines that had undergone pharmacologic demethylation.

Using the same integrative approach we previously reported [2], [3] we were able to identify *MAGEB2* as a novel gene whose transcription in HNSCC is associated with promoter demethylation. We then further corroborated this finding by QUMSP/BSS and RT-PCR in a large cohort of samples (total of n = 166 HNSCC, n = 60 normal mucosa).

*MAGEB2* is a member of the cancer testis antigens, in particularly of the *MAGEB* family located in the last exon of chromosome×which was first reported in 1997 by Lurquin et al [23]. *MAGE-B* genes are a cluster of four human genes in Xp21.3 that are homologous to the *MAGE* genes located in Xq28. The coding regions of the *MAGE-B* genes share about 75% nucleotide identity. They show about 60% identity with those of most *MAGE-A* genes. Except for testis and placenta, no normal tissue was found to express any of the four *MAGE-B* genes. *MAGEB2* gene is localized in the dosage-sensitive sex reversal critical region.

Previous studies have shown that the expression of *MAGE-A* and *MAGE-B* genes in tumors is regulated by promoter methylation [23], [24]. *MAGEB2* expression was shown to be the most abundantly expressed of the *MAGE-B* family, and has been shown to be overexpressed in several tumors types [25], [26]. In this study we show that *MAGEB2* is activated by promoter demethylation in HNSCC.

The *MAGE* family of genes has been considered as potential cancer vaccine targets, having been shown to elicit coordinated humoral and cell mediated responses. Indeed multiple vaccine based clinical trials have been carried out targeting *MAGEA3* and NY-ESO-1 [27], [28]. *MAGEB2* overexpression in tumors seems to be an attractive target for therapy since it could suffice to generate antigenic peptides leading to immunogenicity and thereby rendering them susceptible to vaccination therapy similarly to the *MAGE-A* genes. This would be particularly important in those tumors that do not express any of the *MAGE-A* genes.

Immunohistochemical analysis has revealed that the cancer testis antigens are rarely homogenously expressed in tumors (28). This is in accordance with our findings of heterogeneous *MAGEB2* expression, in HNSCC tumor tissues. They are frequently found only in a relatively small proportion of the cells in a tumor, and it has been suggested that it could be that these antigens serve as markers for cells with stem cell like properties within the tumor [29].

The biological function of the *MAGE* family of genes in both the germ line and tumors has remained poorly understood. Nonetheless, it appears that the *MAGE* genes encode multifunctional regulator molecules that exert a large range of effects. The *MAGE-A* genes have been shown to act as transcriptional repressors [30]. *MAGE-A2* was shown to strongly down regulate *p53* transactivation function, and association between *MAGE-A* expression levels and resistance to etoposide treatment was shown in short- term melanoma cell lines harboring wild-type *p53* [31]. *MAGEC1* and *MAGEA3* were shown to play an important role in promoting survival by reducing the rate of apoptosis [32], [33]. Recently it was shown that *MAGE-A* suppresses the *p53* transcriptional program during tumor development [34].

In contrast, whether the *MAGE-B* genes have a role in tumor development, proliferation and growth has not been reported yet. Since we found that *MAGEB2* was overexpressed almost exclusively in tumors as a result of promoter demethylation we further sought whether it may have a role as an oncogene promoting tumor growth and development. We consequently found that forced expression of *MAGEB2* in minimally transformed oral keratinocytes lead to 179% (+/−36%) increase in growth 72 h post transient transfection respectively, compared to identical mock treated cell lines. We were not able to notice any definitive growth promoting effects on the HNSCC cell lines we tested. Additional studies are needed to fully substantiate this finding and elucidate the exact role of MAGEB2 in HNSCCs..

To summarize, in this present study we show that by using an integrative analysis approach, combining a large cohort of HNSCC and normal mucosa tissue expression arrays data and pharmacologic de-methylation re-expression arrays data from normal oral keratinocyte cell lines and a validation process on a large cohort of primary HNSCC tumor tissues and normal mucosa tissues, we were able to identify *MAGEB2* as activated by promoter demethylation in HNSCC and demonstrates growth promoting effects on a minimally transformed oral keratinocye cell line Further research is needed to completely elucidate the functions and role of *MAGEB2* in tumor development and whether it could be exploited as a target for therapy in HNSCC.

## Supporting Information

Figure S1 **COPA GRAPHS of selected genes.**
(TIF)

Figure S2 **Upfold regulation of candidate genes after treatment 5-aza/TSA in cell line OKF6-T1.** The numbers on the Y axis are the ratio between post and pre treatment values.
(TIF)

Figure S3 **Bisulfite sequencing and RT-PCR results of primary HNSCC tissue samples and of normal mucosa controls.**
(TIF)

Figure S4 **RT PCR Expression Level Data- NOKSI and O22 Transfected Cell Lines.**
(TIF)

Table S1 **RtPCR Primer Sequences.**
(DOCX)

Table S2 **QUMSP Primer and Probe Sequences.**
(DOCX)

Table S3 **Significant genes following primary integrative analysis.**
(DOCX)

Table S4 **Top ranked 178 genes.**
(DOCX)

Table S5 **Top 96 genes.**
(DOCX)

Table S6 **QUMSP unmethylation levels (gene of interest/bactin ×100) for 4 tested genes in primary HNSCC tumor tissues and in normal mucosa (UPPP).**
(DOCX)

Table S7 **Normalized RT PCR expression levels of MAGEB2 on primary HNSCC tumor tissues and normal mucosa tissues.**
(DOCX)
