# Supplementary figures and images for: MAGEB2 is Activated by Promoter Demethylation in Head and Neck Squamous Cell Carcinoma
Source: PLoS One. 2012 Sep 24;7(9):e45534. doi: 10.1371/journal.pone.0045534 (PMC3454438; doi:10.1371/journal.pone.0045534)

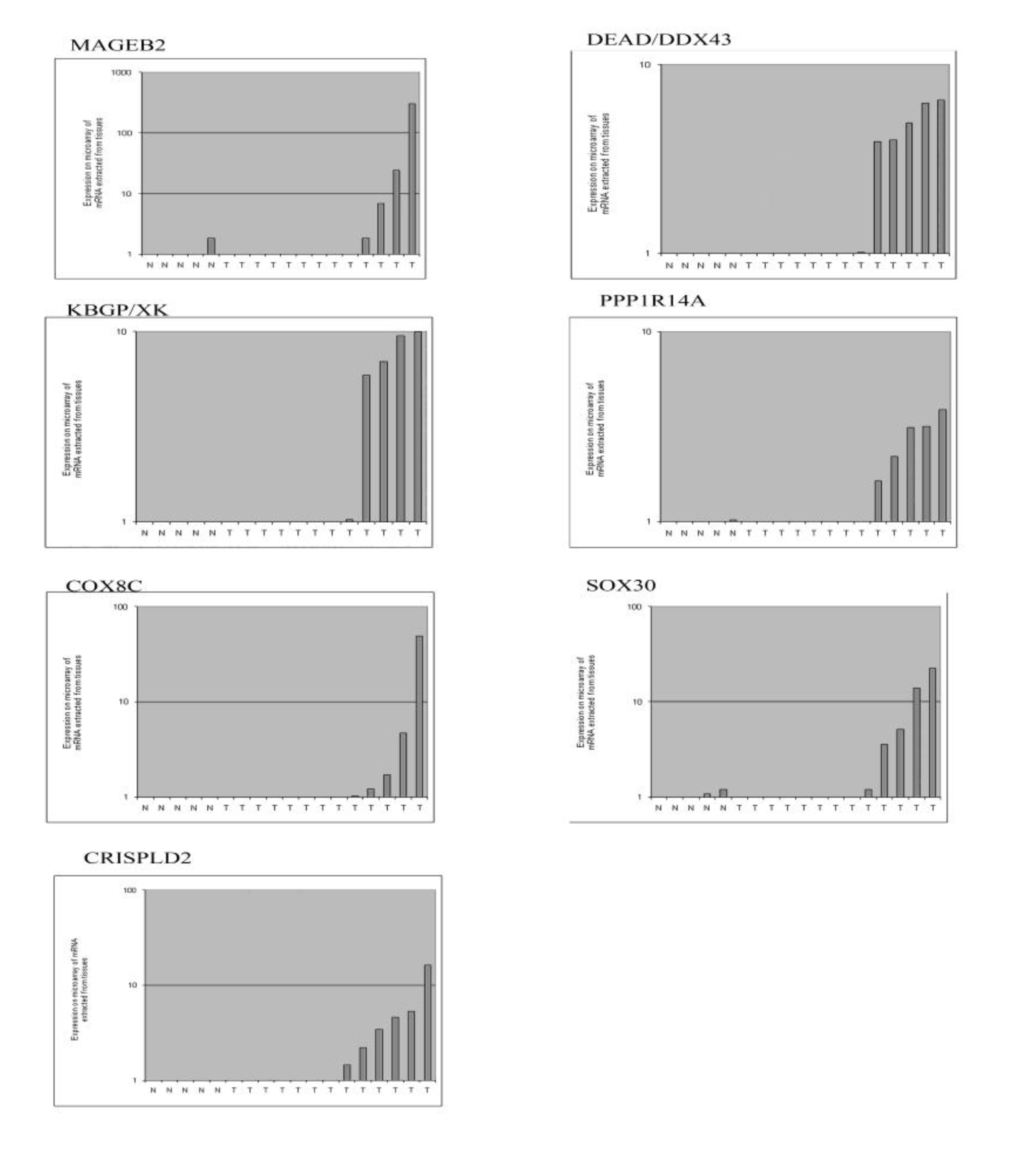

Supplement: Figure S1 — COPA GRAPHS of selected genes. (TIF) [file pone.0045534.s001.tif]

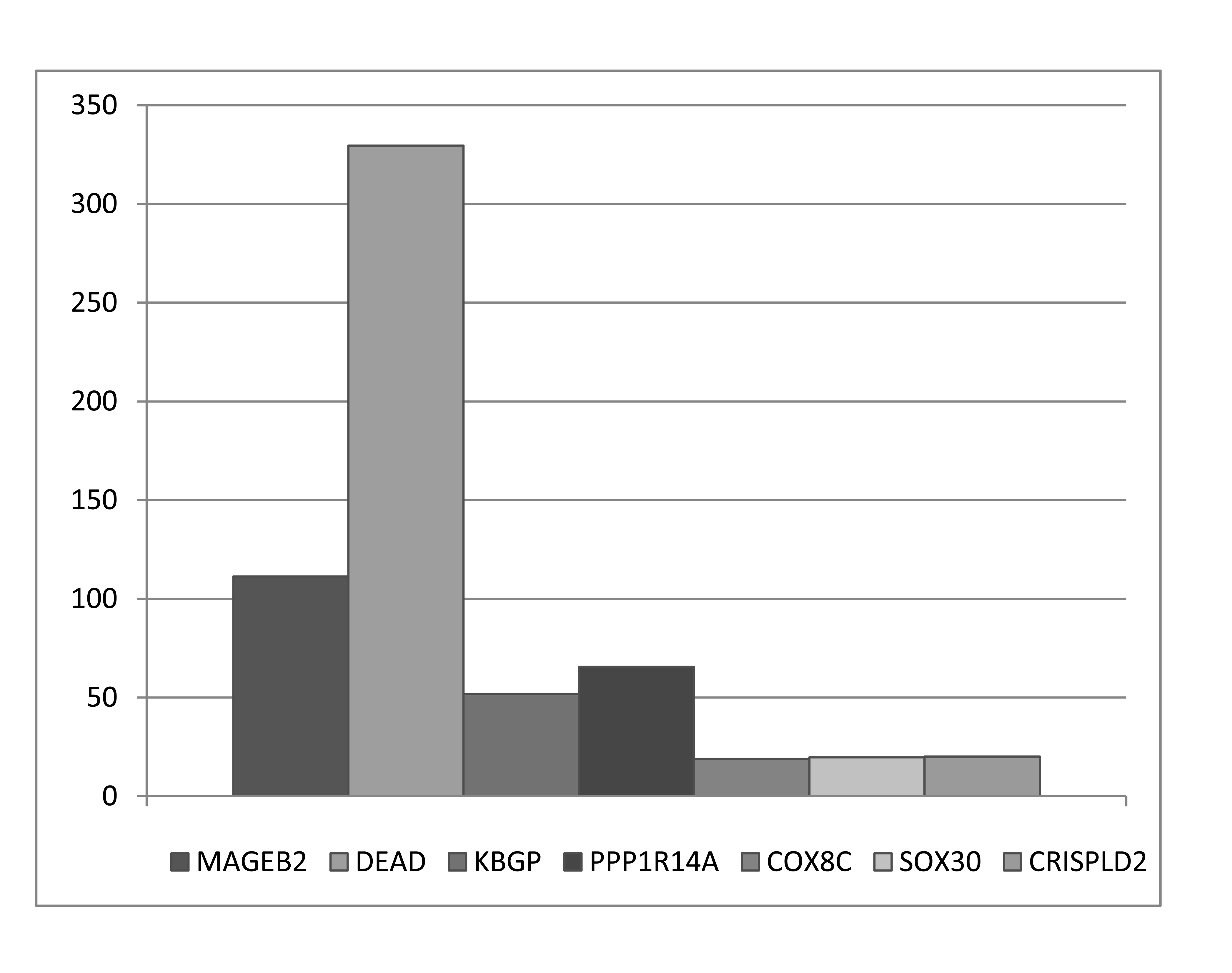

Supplement: Figure S2 — Upfold regulation of candidate genes after treatment 5-aza/TSA in cell line OKF6-T1. The numbers on the Y axis are the ratio between post and pre treatment values. (TIF) [file pone.0045534.s002.tif]

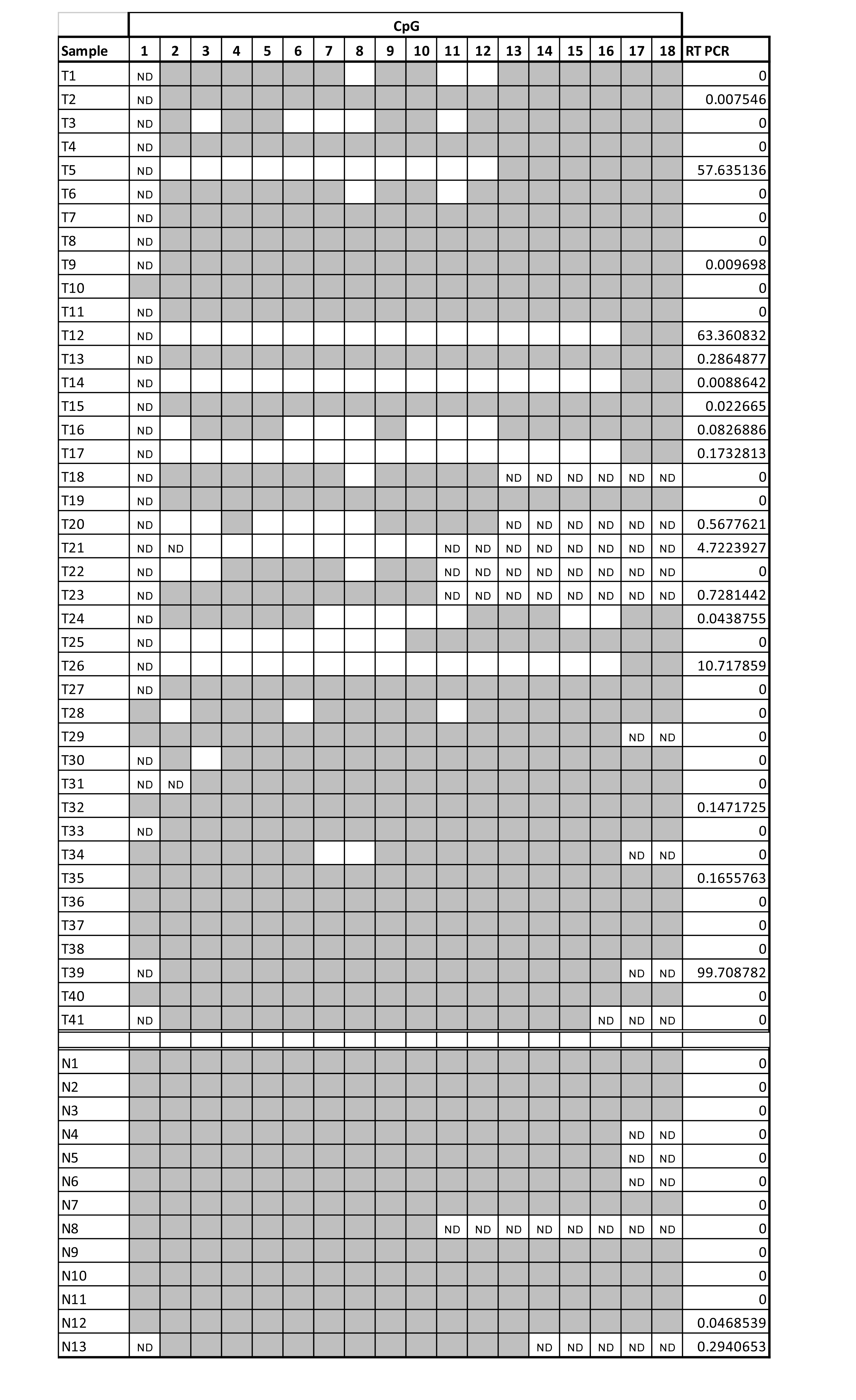

Supplement: Figure S3 — Bisulfite sequencing and RT-PCR results of primary HNSCC tissue samples and of normal mucosa controls. (TIF) [file pone.0045534.s003.tif]

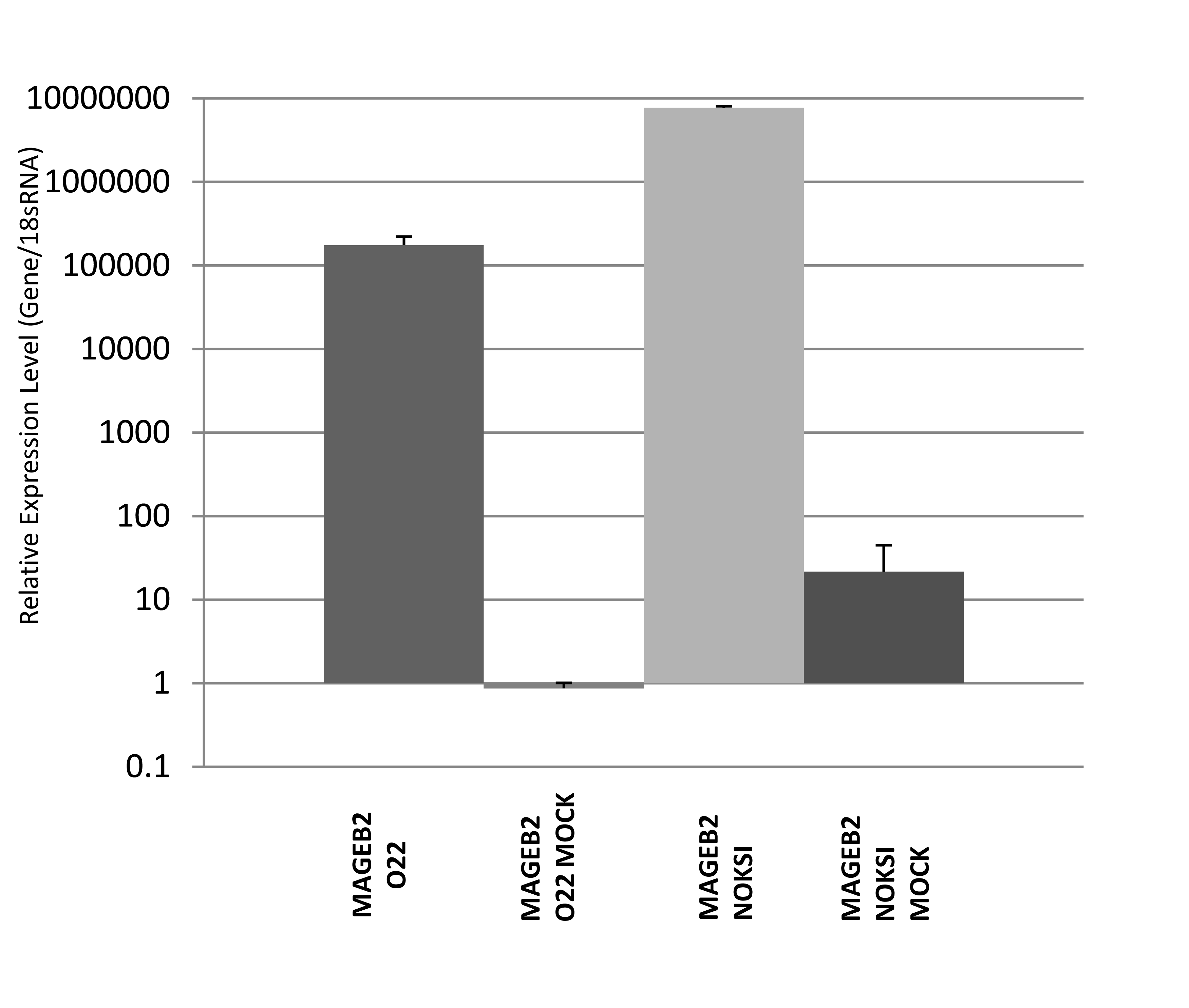

Supplement: Figure S4 — RT PCR Expression Level Data- NOKSI and O22 Transfected Cell Lines. (TIF) [file pone.0045534.s004.tif]
