# Supplementary material for: MAGEB2 is Activated by Promoter Demethylation in Head and Neck Squamous Cell Carcinoma
Source: PLoS One. 2012 Sep 24;7(9):e45534. doi: 10.1371/journal.pone.0045534 (PMC3454438; doi:10.1371/journal.pone.0045534)
Supplement: Table S1 — RtPCR Primer Sequences. (DOCX) [file pone.0045534.s005.docx]

**Suppl Table 1- RtPCR Primer Sequences**

| **Gene** | **Forward Primer** | **Reverse Primer** |
| --- | --- | --- |
| MAGEB2 | CCCGAGCGAGTGTAGGGGGT | CTCATCTCGGGCCTTGCGGC |
| DEAD/DDX43 | ATGATTGGAGGGTTGCCTCT | GGCCTTCCTTGAGGTCTTTC |
| KBGP/XK | CTTCCTGTGGAGGAGCTTTG | AGAACTGACCAGGCTCAGGA |
| 18S | GGATCCATTGGAGGGCAAGT | CGGTGGCTCGCCTCG |
