# Supplementary material for: MAGEB2 is Activated by Promoter Demethylation in Head and Neck Squamous Cell Carcinoma
Source: PLoS One. 2012 Sep 24;7(9):e45534. doi: 10.1371/journal.pone.0045534 (PMC3454438; doi:10.1371/journal.pone.0045534)
Supplement: Table S2 — QUMSP Primer and Probe Sequences. (DOCX) [file pone.0045534.s006.docx]

**Suppl Table 2. QUMSP Primer and Probe Sequences**

| **Gene name** | **Forward Primer** | **Reverse Primer** | **Probe(FAM-TAMRA)** |
| --- | --- | --- | --- |
| **DEAD** | ATTGGGAAAGTTTTGTTTATAATGT | CCAACTCTACACCACCTAACCATA | CAC TCT CTA TCC CAC CCA CTT CAC AC |
| **KBGP** | TTAGTTTTAGAGTTTAGGTTGGTTGG | TATAACTATAACAACAACAACCATT | CAC TCC CAC ACT CTA CAC AAC CCA CC |
| **MAGEB2** | GTGGAGATGAAGGGATTAGTAATGT | AAAATCAAAATAACTCACATCCAAC | ACT AAC AAC TAA ACA CTA CCA AAC AT |
