# Supplementary material for: MAGEB2 is Activated by Promoter Demethylation in Head and Neck Squamous Cell Carcinoma
Source: PLoS One. 2012 Sep 24;7(9):e45534. doi: 10.1371/journal.pone.0045534 (PMC3454438; doi:10.1371/journal.pone.0045534)
Supplement: Table S3 — Significant genes following primary integrative analysis. (DOCX) [file pone.0045534.s007.docx]

**Supplementary Table 3- Significant genes following primary integrative analysis**

| Gene Name | Accesion # |
| --- | --- |
| melanoma antigen family A, 4 | AW438674 |
| melanoma antigen family A, 6 | U10691 |
| dehydrogenase/reductase (SDR family) member 2 | AK000345 |
| matrix metalloproteinase 13 (collagenase 3) /// matrix metalloproteinase 13 (collagenase 3) | NM_002427 |
| hypothetical protein LOC254848 | BC043614 |
| melanoma antigen family A, 3 | BC000340 |
| homeo box HB9 | AI738662 |
| melanoma antigen family A, 12 | BC003408 |
| fatty acid binding protein 4, adipocyte | NM_001442 |
| FYVE, RhoGEF and PH domain containing 2 | AW516510 |
| cytokeratin type II | NM_004693 |
| heat shock 70kDa protein 2 | U56725 |
| plasminogen activator, tissue | NM_000930 |
| Full-length cDNA clone CS0DI027YJ05 of Placenta Cot 25-normalized of Homo sapiens (human) | N30878 |
| solute carrier family 8 (sodium-calcium exchanger), member 2 | AI127885 |
| melanoma antigen family A, 11 | BC004479 |
| melanoma antigen family B, 2 | NM_002364 |
| astrotactin 2 | AF116574 |
| cysteine-rich secretory protein LCCL domain containing 2 | AL136861 |
| Full length insert cDNA clone YB44H10 | AF147356 |
| apolipoprotein C-I | NM_001645 |
| dehydrogenase/reductase (SDR family) member 2 | NM_005794 |
| contactin associated protein-like 2 | AC005378 |
| transketolase-like 1 | Z49258 |
| distal-less homeo box 2 | NM_004405 |
| RAB3B, member RAS oncogene family | AU156710 |
| ornithine decarboxylase 1 | NM_002539 |
| keratin, hair, basic, 6 (monilethrix) | X99142 |
| chromosome 6 open reading frame 148 | NM_030568 |
| ubiquitin carboxyl-terminal esterase L1 (ubiquitin thiolesterase) | NM_004181 |
| Transcribed locus, moderately similar to NP_055301.1 neuronal thread protein AD7c-NTP [Homo sapiens] | AA922273 |
| oxoglutarate dehydrogenase-like | NM_018245 |
| Hypothetical protein MGC39606 | AA565509 |
| collagen, type IX, alpha 3 | NM_001853 |
| collagen triple helix repeat containing 1 | AA584310 |
| CDNA clone IMAGE:5301169, partial cds | BM479034 |
| DnaJ (Hsp40) homolog, subfamily C, member 6 | AV729634 |
| KIPV467 | W69083 |
| insulin growth factor-like family member 1 | AA195677 |
| paternally expressed 10 | AL582836 |
| retinol dehydrogenase 12 (all-trans and 9-cis) | AI796235 |
| tripartite motif-containing 9 | AF220036 |
| Hypothetical protein FLJ13744 | NM_025011 |
| hypothetical protein FLJ90166 | BG326592 |
| transmembrane protein SHREW1 | NM_018836 |
| paternally expressed 10 | BE858180 |
| fibulin 2 | NM_001998 |
| keratin, hair, basic, 1 | NM_002281 |
| RAB3B, member RAS oncogene family | BC005035 |
| Hypothetical protein MGC42174 | AL832765 |
| cysteine and glycine-rich protein 2 | U46006 |
| dynamin 3 | AI631915 |
| protein tyrosine phosphatase, receptor type, f polypeptide (PTPRF), interacting protein (liprin), alpha 1 | U22815 |
| prion protein 2 (dublet) | AL133396 |
| chondroitin sulfate proteoglycan 5 (neuroglycan C) | AF059274 |
| Solute carrier family 7 (cationic amino acid transporter, y+ system), member 8 | AL365343 |
| transmembrane protein SHREW1 | AA835004 |
| gb:BF111214 /DB_XREF=gi:10940904 /DB_XREF=7n44e07.x1 /CLONE=IMAGE:3567468 /FEA=EST /CNT=5 /TID=Hs.128138.0 /TIER=ConsEnd /STK=5 /UG=Hs.128138 /UG_TITLE=ESTs, Weakly similar to ATS1_HUMAN ADAM-TS 1 PRECURSOR (H.sapiens) | BF111214 |
| carnitine palmitoyltransferase 1C | AL565745 |
| LOC440570 | AI733297 |
| trophoblast-derived noncoding RNA | AV659198 |
| protein phosphatase 1, regulatory (inhibitor) subunit 14A | AA156998 |
| dipeptidylpeptidase 4 (CD26, adenosine deaminase complexing protein 2) | M80536 |
| G protein-coupled receptor 54 | AI819198 |
| Solute carrier family 7 (cationic amino acid transporter, y+ system), member 8 | AL365343 |
| hairy/enhancer-of-split related with YRPW motif 2 | AF232238 |
| interleukin 8 | AF043337 |
| interleukin 1 receptor-like 1 | NM_003856 |
| Transcribed locus | AA876179 |
| glutamate receptor, ionotropic, N-methyl D-aspartate 1 | NM_007327 |
| dispatched homolog 2 (Drosophila) | AB051529 |
| Ras-induced senescence 1 | BF062629 |
| disabled homolog 1 (Drosophila) | NM_021080 |
| kinesin family member 26A | AK026406 |
| RAS, dexamethasone-induced 1 | AF069506 |
| hypothetical gene supported by AK123741 | BF109906 |
| interleukin 8 | NM_000584 |
| Transcribed locus | BE670161 |
| hypothetical gene supported by AK125122 | AA129774 |
| placenta-specific 1 | NM_021796 |
| Full-length cDNA clone CS0DJ013YP06 of T cells (Jurkat cell line) Cot 10-normalized of Homo sapiens (human) | H49805 |
| chemokine (C-X-C motif) ligand 11 | AF002985 |
| cadherin, EGF LAG seven-pass G-type receptor 3 (flamingo homolog, Drosophila) | NM_001407 |
| similar to mouse 1700027M21Rik gene | AI964053 |
| KIAA1937 protein | AK093300 |
| transcription factor AP-2 beta (activating enhancer binding protein 2 beta) | NM_003221 |
| interleukin 6 (interferon, beta 2) | NM_000600 |
| arachidonate 15-lipoxygenase, second type | NM_001141 |
| Guanylate binding protein 5 | BG545653 |
| peptide YY, 2 (seminalplasmin) | NM_021093 |
| gb:AI810266 /DB_XREF=gi:5396832 /DB_XREF=wb86h07.x1 /CLONE=IMAGE:2312605 /FEA=EST /CNT=12 /TID=Hs.130853.0 /TIER=ConsEnd /STK=5 /UG=Hs.130853 /UG_TITLE=ESTs | AI810266 |
| insulin-like growth factor binding protein 3 | BF340228 |
| SRY (sex determining region Y)-box 30 | NM_007017 |
| chemokine (C-X-C motif) ligand 3 | NM_002090 |
| transglutaminase 2 (C polypeptide, protein-glutamine-gamma-glutamyltransferase) | M98478 |
| cysteine and glycine-rich protein 2 | NM_001321 |
| SPANX family, member B1 /// SPANX family, member B2 | NM_013453 |
| ras homolog gene family, member B | AI263909 |
| RASD family, member 2 | AF279143 |
| Homo sapiens, clone IMAGE:3891572, mRNA | BC009533 |
| trophoblast-derived noncoding RNA | AU155361 |
| insulin-like growth factor binding protein 3 | M31159 |
| Homo sapiens, clone IMAGE:4822964, mRNA | BC034800 |
| Transcribed locus, moderately similar to XP_517454.1 similar to hypothetical protein MGC45438 [Pan troglodytes] | BF056092 |
| bone marrow stromal cell antigen 2 | NM_004335 |
| collagen, type II, alpha 1 (primary osteoarthritis, spondyloepiphyseal dysplasia, congenital) | X16468 |
| myosin, light polypeptide kinase /// myosin, light polypeptide kinase | NM_005965 |
| collagen, type V, alpha 3 | NM_015719 |
| Suppression of tumorigenicity 7 like | BF961733 |
| Exostoses (multiple) 1 | BC017944 |
| collagen, type VIII, alpha 2 | AI806793 |
| intercellular adhesion molecule 1 (CD54), human rhinovirus receptor | AI608725 |
| nanos homolog 1 (Drosophila) | AW970089 |
| crystallin, alpha B | AF007162 |
| Xg blood group (pseudoautosomal boundary-divided on the X chromosome) | AF380356 |
| Stanniocalcin 1 | AW003173 |
| peptidylprolyl isomerase (cyclophilin)-like 2 | NM_014337 |
| metastasis associated lung adenocarcinoma transcript 1 (non-coding RNA) | AF132202 |
| DEAD (Asp-Glu-Ala-Asp) box polypeptide 43 | NM_018665 |
| interleukin 20 | AF224266 |
| Kell blood group precursor (McLeod phenotype) | NM_021083 |
| keratin, hair, acidic, 4 | NM_021013 |
| G protein-coupled receptor 24 | AI934819 |
| DNA (cytosine-5-)-methyltransferase 3 beta | NM_006892 |
| hypothetical LOC388206 | AI762077 |
| protocadherin 10 | AI640307 |
| Similar to hypothetical protein FLJ20296 | AK098125 |
| potassium channel, subfamily K, member 15 | NM_022358 |
| S100 calcium binding protein A7-like 1 | AJ243672 |
| endothelin 1 | BC036851 |
| CDNA FLJ11397 fis, clone HEMBA1000622 | AU144005 |
| small proline-rich protein 2G | AA456642 |
| relaxin 3 | AB076563 |
| neuronal pentraxin II | U26662 |
| TSC22 domain family 2 | AF201291 |
| neurexophilin 4 | AI933199 |
| sodium channel, voltage-gated, type I, beta | NM_001037 |
| ras homolog gene family, member B | BI668074 |
| v-maf musculoaponeurotic fibrosarcoma oncogene homolog B (avian) | NM_005461 |
| Transmembrane protein 41B | N64760 |
| transglutaminase 2 (C polypeptide, protein-glutamine-gamma-glutamyltransferase) | AL031651 |
| SMAD, mothers against DPP homolog 6 (Drosophila) | NM_005585 |
| neuronal guanine nucleotide exchange factor | AV703769 |
| pentraxin-related gene, rapidly induced by IL-1 beta | NM_002852 |
| Multiple C2-domains with two transmembrane regions 1 | BG250585 |
| chemokine (C-X-C motif) ligand 11 | AF030514 |
| Full open reading frame cDNA clone RZPDo834C0824D for gene HIST2H4, histone 2, H4; complete cds, incl. stopcodon | AI828075 |
| dual specificity phosphatase 9 | NM_001395 |
| dipeptidylpeptidase 4 (CD26, adenosine deaminase complexing protein 2) | M74777 |
| unc-93 homolog A (C. elegans) | AL021331 |
| doublesex and mab-3 related transcription factor 1 | NM_021951 |
| hypothetical protein FLJ14351 | NM_024732 |
| lumican | NM_002345 |
| forkhead box protein O6 | AI341823 |
| chromosome 9 open reading frame 140 | AW250904 |
| gb:R41431 /DB_XREF=gi:816734 /DB_XREF=yf95c10.s1 /CLONE=IMAGE:30249 /FEA=EST /CNT=4 /TID=Hs.22495.0 /TIER=ConsEnd /STK=4 /UG=Hs.22495 /UG_TITLE=ESTs | R41431 |
| cytochrome c oxidase subunit 8C | AW269746 |
| cytochrome P450, family 24, subfamily A, polypeptide 1 | NM_000782 |
| MRNA; cDNA DKFZp686H1890 (from clone DKFZp686H1890) | AI083557 |
| family with sequence similarity 20, member A | AW291369 |
| prostaglandin-endoperoxide synthase 1 (prostaglandin G/H synthase and cyclooxygenase) | S36219 |
| fatty acid 2-hydroxylase | NM_024306 |
| gb:AI394574 /DB_XREF=gi:4224121 /DB_XREF=tg08c12.x1 /CLONE=IMAGE:2108182 /FEA=EST /CNT=8 /TID=Hs.157123.0 /TIER=Stack /STK=8 /UG=Hs.157123 /UG_TITLE=ESTs | AI394574 |
| coagulation factor II (thrombin) receptor-like 2 | AI378647 |
| lysosomal-associated membrane protein 3 | NM_014398 |
| DKFZP586B0319 protein | AI692879 |
| HORMA domain containing 1 | AL136755 |
| synaptophysin | U93305 |
| ATP-binding cassette, sub-family A (ABC1), member 4 | U88667 |
| hypothetical protein FLJ10052 | BC004888 |
| solute carrier family 7, (cationic amino acid transporter, y+ system) member 13 | AI471866 |
| ADAMTS-like 2 | NM_014694 |
| histone 1, H2bd | AL353759 |
| defensin, beta 1 | U73945 |
| hippocalcin-like 1 | NM_002149 |
| reticulocalbin 3, EF-hand calcium binding domain | AI797684 |
| protein tyrosine phosphatase, receptor type, f polypeptide (PTPRF), interacting protein (liprin), alpha 4 | AK023365 |
| PDZ domain containing 1 | NM_002614 |
| carboxypeptidase A4 | NM_016352 |
| small nuclear RNA activating complex, polypeptide 1, 43kDa | NM_003082 |
| tumor necrosis factor, alpha-induced protein 6 | NM_007115 |
| mago-nashi homolog | NM_018048 |
| hypothetical protein DKFZP761M1511 | AK026748 |
| gb:BC042682.1 /DB_XREF=gi:27502885 /TID=Hs2.434130.1 /CNT=2 /FEA=mRNA /TIER=ConsEnd /STK=1 /UG=Hs.434130 /UG_TITLE=Homo sapiens, clone IMAGE:4823120, mRNA /DEF=Homo sapiens, clone IMAGE:4823120, mRNA. | BC042682 |
| lectin, galactoside-binding, soluble, 7 (galectin 7) | NM_002307 |
| solute carrier family 13 (sodium/sulfate symporters), member 1 | NM_022444 |
| microtubule-associated protein 1B | BG164365 |
| cytochrome P450, family 26, subfamily B, polypeptide 1 | NM_019885 |
| gamma-glutamyltransferase-like activity 1 | NM_004121 |
| interferon, alpha-inducible protein 27 | NM_005532 |
| chromosome 9 open reading frame 11 | BC014307 |
| prostaglandin-endoperoxide synthase 1 (prostaglandin G/H synthase and cyclooxygenase) | NM_000962 |
| v-maf musculoaponeurotic fibrosarcoma oncogene homolog B (avian) | AW135013 |
| Smoothened homolog (Drosophila) | AK024098 |
| ribonuclease P 25kDa subunit | NM_017793 |
| frizzled homolog 9 (Drosophila) | NM_003508 |
| wingless-type MMTV integration site family, member 7B | BE736994 |
| synaptogyrin 3 | NM_004209 |
| prostaglandin-endoperoxide synthase 1 (prostaglandin G/H synthase and cyclooxygenase) | BE613133 |
| suprabasin | AI814274 |
| snail homolog 1 (Drosophila) | NM_005985 |
| Mdm4, transformed 3T3 cell double minute 1, p53 binding protein (mouse) | NM_020128 |
| epiplakin 1 | AL137725 |
| solute carrier family 8 (sodium/calcium exchanger), member 1 | NM_021097 |
| gb:BE467579 /DB_XREF=gi:9513354 /DB_XREF=hz72f06.x1 /CLONE=IMAGE:3213539 /FEA=EST /CNT=5 /TID=Hs.156868.0 /TIER=ConsEnd /STK=4 /UG=Hs.156868 /UG_TITLE=ESTs | BE467579 |
| adenylate cyclase 1 (brain) | AA021120 |
| MRNA; cDNA DKFZp666I029 (from clone DKFZp666I029) | AL833053 |
| calcium and integrin binding family member 2 | NM_006383 |
| transient receptor potential cation channel, subfamily A, member 1 | AA502609 |
| complement component 1, q subcomponent-like 1 | NM_006688 |
| hypothetical protein LOC340529 | BC041956 |
| HLA complex P5 | NM_006674 |
| MRNA; cDNA DKFZp434I1526 (from clone DKFZp434I1526) | AL157460 |
| Transcribed locus, weakly similar to XP_518970.1 similar to GLCCI1 protein [Pan troglodytes] | AI492376 |
| KIAA0251 protein | N70298 |
| gb:AA971131 /DB_XREF=gi:3146421 /DB_XREF=op71g09.s1 /CLONE=IMAGE:1582336 /FEA=EST /CNT=4 /TID=Hs.128647.0 /TIER=ConsEnd /STK=4 /UG=Hs.128647 /UG_TITLE=ESTs | AA971131 |
| histone 1, H2bo | NM_003527 |
| cytochrome c oxidase subunit VIb polypeptide 2 (testis) | NM_144613 |
| Interleukin 12 receptor, beta 2 | R01220 |
| dynamin 1 | AF035321 |
| ets variant gene 7 (TEL2 oncogene) | AF218365 |
| X (inactive)-specific transcript | AV646335 |
| Homo sapiens, clone IMAGE:5165147, mRNA | BC038532 |
| radical S-adenosyl methionine domain containing 2 | AW189843 |
| Hypothetical protein LOC145663 | BC039389 |
| hypothetical protein FLJ20152 | NM_019000 |
| tumor-associated calcium signal transducer 1 | NM_002354 |
| psoriasis susceptibility 1 candidate 2 | NM_014069 |
| neurogranin (protein kinase C substrate, RC3) | NM_006176 |
| epiplakin 1 | AL137725 |
| Transcribed locus | AI093492 |
| A disintegrin-like and metalloprotease (reprolysin type) with thrombospondin type 1 motif, 15 | AI970797 |
| Chromosome 10 open reading frame 56 | BC029259 |
| hypothetical protein LOC286126 | AK023309 |
| cysteine-rich, angiogenic inducer, 61 | AF003114 |
| ATPase, Class VI, type 11B | BC033880 |
| solute carrier family 6 (neurotransmitter transporter, creatine), member 8 | NM_005629 |
| Chromosome 21 open reading frame 2 | U84569 |
| gb:AV652328 /DB_XREF=gi:9873342 /DB_XREF=AV652328 /CLONE=GLCCZC01 /FEA=EST /CNT=7 /TID=Hs.282488.0 /TIER=ConsEnd /STK=3 /UG=Hs.282488 /UG_TITLE=ESTs | AV652328 |
| Brain-derived neurotrophic factor | AJ011597 |
| CD74 antigen (invariant polypeptide of major histocompatibility complex, class II antigen-associated) | K01144 |
| histone 1, H1c | BC002649 |
| intercellular adhesion molecule 1 (CD54), human rhinovirus receptor | NM_000201 |
| gb:AA290605 /DB_XREF=gi:1938867 /DB_XREF=zs45c09.s1 /CLONE=IMAGE:700432 /FEA=EST /CNT=4 /TID=Hs.190002.0 /TIER=ConsEnd /STK=3 /UG=Hs.190002 /UG_TITLE=ESTs | AA290605 |
| Cystatin E/M | AI188465 |
| leucine zipper, putative tumor suppressor 1 | N21184 |
| dickkopf homolog 1 (Xenopus laevis) | NM_012242 |
| leucine zipper protein pseudogene 1 | BF223086 |
| aldo-keto reductase family 1, member C-like 2 | AB040820 |
| keratin associated protein 2-1 /// keratin associated protein 2-4 | BC012486 |
| FLJ35767 protein | BE205922 |
| CDNA FLJ11397 fis, clone HEMBA1000622 | AW962458 |
| gb:AI806174 /DB_XREF=gi:5392740 /DB_XREF=wf06h03.x1 /CLONE=IMAGE:2349845 /FEA=mRNA /CNT=125 /TID=Hs.232068.2 /TIER=Stack /STK=32 /UG=Hs.232068 /LL=6935 /UG_GENE=TCF8 /UG_TITLE=transcription factor 8 (represses interleukin 2 expression) | AI806174 |
| chemokine (C-X-C motif) ligand 1 (melanoma growth stimulating activity, alpha) | NM_001511 |
| histone 1, H2bd | BC002842 |
| armadillo repeat containing, X-linked 2 | NM_014782 |
| Similar to hypothetical protein FLJ25955 | BF515709 |
| calmodulin-like 5 | NM_017422 |
| nuclear receptor interacting protein 3 | NM_020645 |
| hypothetical protein FLJ21511 | NM_025087 |
| Homo sapiens, clone IMAGE:5266022, mRNA | AL163533 |
| Slit homolog 2 (Drosophila) | AI692523 |
| neurturin | AL161995 |
| Full-length cDNA clone CS0DI027YJ20 of Placenta Cot 25-normalized of Homo sapiens (human) | BF060672 |
| gb:W93728 /DB_XREF=gi:1422918 /DB_XREF=zd96a11.s1 /CLONE=IMAGE:357308 /FEA=FLmRNA /CNT=84 /TID=Hs.77890.0 /TIER=Stack /STK=29 /UG=Hs.77890 /LL=2983 /UG_GENE=GUCY1B3 /UG_TITLE=guanylate cyclase 1, soluble, beta 3 /FL=gb:NM_000857.1 | W93728 |
| forkhead box A2 | AB028021 |
| early growth response 4 | NM_001965 |
| ephrin-A3 | AW189015 |
| carbohydrate (N-acetylglucosamine-6-O) sulfotransferase 2 | NM_004267 |
| LIM and cysteine-rich domains 1 | NM_014583 |
| gb:AI494047 /DB_XREF=gi:4395050 /DB_XREF=qz55b08.x1 /CLONE=IMAGE:2030775 /FEA=EST /CNT=6 /TID=Hs.169816.0 /TIER=ConsEnd /STK=0 /UG=Hs.169816 /UG_TITLE=ESTs | AI494047 |
| prostaglandin-endoperoxide synthase 1 (prostaglandin G/H synthase and cyclooxygenase) | NM_000962 |
| early growth response 2 (Krox-20 homolog, Drosophila) | NM_000399 |
| CDNA FLJ43552 fis, clone PROST2017972 | H41121 |
| Transmembrane protein 41B | AU153138 |
| histone 2, H2be | NM_003528 |
| Interleukin 17D | AI669535 |
| Hypothetical protein DKFZp434D2328 | AV649411 |
| pyrimidinergic receptor P2Y, G-protein coupled, 6 | NM_004154 |
| membrane progestin receptor gamma | AI934557 |
| hypothetical protein LOC285593 | BC033564 |
| histone 1, H2ac | AL353759 |
| early B-cell factor 3 | BF592034 |
| actin binding LIM protein family, member 2 | AI240129 |
| transmembrane protein 2 | NM_013390 |
| hypothetical LOC387763 | AW276078 |
| Hypothetical protein FLJ37874 | BG438112 |
| PHD finger protein 21B | AK092243 |
| stanniocalcin 1 | AI300520 |
| Chromosome 21 open reading frame 86 | AV705934 |
| F-box and WD-40 domain protein 8 | AW136338 |
| solute carrier family 12 (potassium/chloride transporters), member 8 | NM_024628 |
| NADH dehydrogenase (ubiquinone) 1, subcomplex unknown, 1, 6kDa | AK023115 |
| mesenchyme homeo box 2 (growth arrest-specific homeo box) | NM_005924 |
| Ras interacting protein 1 | NM_017805 |
| Tripartite motif-containing 37 | BC011742 |
| metastasis associated lung adenocarcinoma transcript 1 (non-coding RNA) | AW005982 |
| centaurin, alpha 2 | AI761520 |
| TYRO protein tyrosine kinase binding protein | NM_003332 |
| glycine amidinotransferase (L-arginine:glycine amidinotransferase) | NM_001482 |
| hypothetical protein MGC14816 | AL832694 |
| serine (or cysteine) proteinase inhibitor, clade I (neuroserpin), member 1 | NM_005025 |
| Down syndrome critical region gene 8 | AA770014 |
| interleukin 11 | NM_000641 |
| C-type lectin domain family 4, member M | AF245219 |
| POU domain, class 2, transcription factor 3 | NM_014352 |
| hypothetical protein FLJ21511 | NM_025087 |
| interleukin 1 family, member 5 (delta) | AF216693 |
| myeloid/lymphoid or mixed-lineage leukemia 2 | AF010404 |
| hypothetical protein LOC339442 | BC042675 |
| IQ motif containing F3 | AW958766 |
| plexin D1 | AL575403 |
| Tripartite motif-containing 8 | BF115135 |
| insulin-like growth factor binding protein 2, 36kDa | NM_000597 |
| Hypothetical protein LOC201175 | AI972838 |
| potassium channel, subfamily T, member 1 | BC035730 |
| TBC1 domain family, member 16 | W74640 |
| pregnancy specific beta-1-glycoprotein 3 | NM_021016 |
| GATA binding protein 2 | AL563460 |
| Glutamate dehydrogenase 1 | AF090918 |
| MRNA; cDNA DKFZp686P24158 (from clone DKFZp686P24158) | AI653960 |
| gb:M81780 /DB_XREF=gi:972768 /FEA=DNA_1 /CNT=1 /TID=Hs.247961.0 /TIER=ConsEnd /STK=0 /UG=Hs.247961 /UG_TITLE=Homo sapiens acid sphingomyelinase (SMPD1) gene, complete cds, ORFs 1-3, complete cdss /DEF=Homo sapiens acid sphingomyelinase (SMPD1) gene, compl | M81780 |
| G protein-coupled receptor 17 | NM_005291 |
| regulating synaptic membrane exocytosis 3 | NM_014747 |
| tigger transposable element derived 3 | N64757 |
| Brain-derived neurotrophic factor | AJ011601 |
| hypothetical protein LOC154761 | AI928764 |
| Integrin, beta 2 (antigen CD18 (p95), lymphocyte function-associated antigen 1; macrophage antigen 1 (mac-1) beta subunit) | BE467566 |
| hypothetical protein LOC129607 | AI742057 |
| nuclear factor (erythroid-derived 2), 45kDa | L13974 |
| MAM domain containing glycosylphosphatidylinositol anchor 1 | AF087987 |
| gb:AL080112.1 /DB_XREF=gi:5262539 /FEA=mRNA /CNT=1 /TID=Hs.332731.0 /TIER=ConsEnd /STK=0 /UG=Hs.332731 /UG_TITLE=Homo sapiens mRNA; cDNA DKFZp586H0722 (from clone DKFZp586H0722) /DEF=Homo sapiens mRNA; cDNA DKFZp586H0722 (from clone DKFZp586H0722). | AL080112 |
| frizzled homolog 8 (Drosophila) | AL121749 |
| Chromosome 9 open reading frame 85 | AI668598 |
| gb:N33403 /DB_XREF=gi:1153802 /DB_XREF=yy41d10.s1 /CLONE=IMAGE:273811 /FEA=EST /CNT=4 /TID=Hs.143764.0 /TIER=ConsEnd /STK=3 /UG=Hs.143764 /UG_TITLE=ESTs, Weakly similar to unknown (H.sapiens) | N33403 |
| gb:AI820802 /DB_XREF=gi:5439881 /DB_XREF=qe74g07.x5 /CLONE=IMAGE:1744764 /FEA=EST /CNT=8 /TID=Hs.246358.0 /TIER=ConsEnd /STK=5 /UG=Hs.246358 /UG_TITLE=ESTs, Weakly similar to T32250 hypothetical protein T15B7.3 - Caenorhabditis elegans (C.elegans) | AI820802 |
| Asparagine synthetase | BF224349 |
| gb:AA779333 /DB_XREF=gi:2838664 /DB_XREF=zj63a05.s1 /CLONE=IMAGE:454928 /FEA=EST /CNT=5 /TID=Hs.20158.0 /TIER=ConsEnd /STK=0 /UG=Hs.20158 /UG_TITLE=ESTs, Weakly similar to S34159 transcription elongation factor IIS (H.sapiens) | AA779333 |
| protein kinase, cAMP-dependent, catalytic, alpha | NM_002730 |
| KIAA1199 | AB033025 |
| lipase, endothelial | NM_006033 |
| stanniocalcin 1 | NM_003155 |
| thiopurine S-methyltransferase | BF196891 |
| left-right determination factor 2 | NM_003240 |
| MRNA; cDNA DKFZp564E233 (from clone DKFZp564E233) | AL049260 |
| hypothetical LOC388727 | AI382195 |
| histone 1, H3h | NM_003536 |
| homeo box A4 | NM_002141 |
| engrailed homolog 1 | NM_001426 |
| hypothetical protein FLJ10618 | AL136803 |
| calcium regulated heat stable protein 1, 24kDa | AL575747 |
| germ cell-less homolog 1 (Drosophila) /// germ cell-less homolog 1 (Drosophila)-like | AF198534 |
| gb:BC006164.1 /DB_XREF=gi:13544062 /FEA=FLmRNA /CNT=1 /TID=HsAffx.900886.997 /TIER=FL /STK=0 /DEF=Homo sapiens, clone MGC:13219, mRNA, complete cds. /PROD=Unknown (protein for MGC:13219) /FL=gb:BC006164.1 | BC006164 |
| transmembrane protein 37 | BI825302 |
| platelet-activating factor receptor /// platelet-activating factor receptor | M80436 |
| Transcribed locus | AW955612 |
| growth arrest and DNA-damage-inducible, gamma interacting protein 1 | BF303939 |
| Nuclear receptor interacting protein 3 | AJ400877 |
| mesoderm specific transcript homolog (mouse) | NM_002402 |
| epiplakin 1 /// epiplakin 1 | NM_031308 |
| gb:NM_018896.1 /DB_XREF=gi:9256522 /GEN=CACNA1G /FEA=FLmRNA /CNT=2 /TID=Hs.194746.0 /TIER=FL /STK=0 /UG=Hs.194746 /LL=8913 /DEF=Homo sapiens calcium channel, voltage-dependent, alpha 1G subunit (CACNA1G), mRNA. /PROD=calcium channel, voltage-dependent, al | NM_018896 |
| oxysterol binding protein 2 | BE501253 |
| Fer3-like (Drosophila) | AF517122 |
| Zinc finger protein 7 (KOX 4, clone HF.16) | AK022466 |
| alanyl (membrane) aminopeptidase (aminopeptidase N, aminopeptidase M, microsomal aminopeptidase, CD13, p150) | NM_001150 |
| SEC15-like 2 (S. cerevisiae) | BF847120 |
| heat shock 70kDa protein 12A | AB007877 |
| Complement component 1, q subcomponent-like 1 | AW026646 |
| phosphoglucomutase 2-like 1 | AA736452 |
| immunoglobulin superfamily, member 4C | BE645821 |
| Low density lipoprotein receptor (familial hypercholesterolemia) | M28219 |
| gb:BE894882 /DB_XREF=gi:10357716 /DB_XREF=601434066F1 /CLONE=IMAGE:3919073 /FEA=EST /CNT=9 /TID=Hs.140237.0 /TIER=ConsEnd /STK=0 /UG=Hs.140237 /UG_TITLE=ESTs, Weakly similar to ALU1_HUMAN ALU SUBFAMILY J SEQUENCE CONTAMINATION WARNING ENTRY (H.sapiens) | BE894882 |
| hypothetical protein FLJ23451 | BC029359 |
| sushi domain containing 2 | Z92546 |
| growth factor independent 1B (potential regulator of CDKN1A, translocated in CML) | AI097490 |
| solute carrier family 2 (facilitated glucose transporter), member 3 | BE550486 |
| hypothetical protein FLJ12748 /// hypothetical protein FLJ12748 | BC006434 |
| glycine amidinotransferase (L-arginine:glycine amidinotransferase) | X86401 |
| heparanase | AF155510 |
| hippocalcin-like 1 | BE617588 |
| Homo sapiens, clone IMAGE:5167029, mRNA | BC031668 |
| PRKC, apoptosis, WT1, regulator | BF732462 |
| gb:BF940313 /DB_XREF=gi:12357633 /DB_XREF=7o43h08.x1 /CLONE=IMAGE:3577190 /FEA=EST /CNT=4 /TID=Hs.122115.1 /TIER=ConsEnd /STK=4 /UG=Hs.122115 /UG_TITLE=ESTs | BF940313 |
| insulin induced gene 1 | BG292233 |
| solute carrier family 2 (facilitated glucose transporter), member 3 /// solute carrier family 2 (facilitated glucose transporter), member 14 | AA778684 |
| KIAA1571 protein | AV734793 |
| Cell adhesion molecule with homology to L1CAM (close homolog of L1) | BC029457 |
| interleukin 32 /// interleukin 32 | NM_004221 |
| chromosome 16 open reading frame 45 | BE299456 |
| chemokine (C-X-C motif) ligand 6 (granulocyte chemotactic protein 2) | NM_002993 |
| hypothetical protein LOC339456 | BE552414 |
| bone morphogenetic protein 2 | AA583044 |
| KIAA0493 protein | AB007962 |
| gb:AL360145.1 /DB_XREF=gi:8919170 /TID=Hs2.271566.1 /CNT=6 /FEA=mRNA /TIER=ConsEnd /STK=0 /UG=Hs.271566 /UG_TITLE=Homo sapiens mRNA full length insert cDNA clone EUROIMAGE 839551. /DEF=Homo sapiens mRNA full length insert cDNA clone EUROIMAGE 839551. | AL360145 |
| T-cell lymphoma invasion and metastasis 1 | U90902 |
| frizzled homolog 5 (Drosophila) | NM_003468 |
| Wolfram syndrome 1 (wolframin) | BC030130 |
| dynein light chain 2 | AA401429 |
| gb:AW005530 /DB_XREF=gi:5854308 /DB_XREF=wz87c11.x1 /CLONE=IMAGE:2565812 /FEA=EST /CNT=5 /TID=Hs.189402.0 /TIER=ConsEnd /STK=5 /UG=Hs.189402 /UG_TITLE=ESTs | AW005530 |
| Hypothetical gene supported by AK094796 | AW614589 |
| selenoprotein X, 1 | NM_016332 |
| gb:AL121723 /DB_XREF=gi:7406637 /FEA=DNA /CNT=1 /TID=Hs.283835.0 /TIER=ConsEnd /STK=0 /UG=Hs.283835 /UG_TITLE=Human DNA sequence from clone RP5-854E16 on chromosome 20 Contains a Soggy-1 (SGY-1) pseudogene, a pseudogene similar to rat CDK5 activator-bindi | AL121723 |
| Williams-Beuren syndrome chromosome region 17 | AI972623 |
| frizzled homolog 10 (Drosophila) | NM_007197 |
| paraneoplastic antigen | AI422335 |
| gb:NM_173594.1 /DB_XREF=gi:27734784 /TID=Hs2.270868.1 /CNT=6 /FEA=FLmRNA /TIER=FL /STK=1 /LL=283372 /UG_GENE=FLJ25613 /UG=Hs.270868 /UG_TITLE=hypothetical protein FLJ25613 /DEF=Homo sapiens hypothetical protein FLJ25613 (FLJ25613), mRNA. /FL=gb:NM_173594. | NM_173594 |
| hypothetical protein LOC284578 | BC043647 |
| BRCA1 associated protein | AI734156 |
| Transcribed locus, moderately similar to XP_510104.1 similar to hypothetical protein FLJ25224 [Pan troglodytes] | AW451426 |
| SH2 domain protein 2A | NM_003975 |
| gb:BC043385.1 /DB_XREF=gi:27694545 /TID=Hs2.178290.1 /CNT=5 /FEA=mRNA /TIER=ConsEnd /STK=0 /UG=Hs.178290 /UG_TITLE=Homo sapiens, clone IMAGE:5246577, mRNA /DEF=Homo sapiens, clone IMAGE:5246577, mRNA. | BC043385 |
| synaptonemal complex protein 3 | AF492003 |
| pygopus homolog 1 (Drosophila) | AL049925 |
| Insulin-like growth factor binding protein 1 | AV651627 |
| metastasis associated lung adenocarcinoma transcript 1 (non-coding RNA) | BG534952 |
| chemokine (C-C motif) ligand 26 | AF096296 |
| AT rich interactive domain 5B (MRF1-like) | BU171496 |
| elastin microfibril interfacer 2 /// elastin microfibril interfacer 2 | AF270513 |
| membrane protein, palmitoylated 1, 55kDa | NM_002436 |
| hypothetical protein FLJ30525 | BE502436 |
| dihydropyrimidine dehydrogenase | BC008379 |
| Transcribed locus, weakly similar to NP_689672.2 hypothetical protein MGC45438 [Homo sapiens] | BF508702 |
| chromosome 21 open reading frame 123 | AK096071 |
| Transcribed locus, moderately similar to XP_510104.1 similar to hypothetical protein FLJ25224 [Pan troglodytes] | BE551193 |
| EGF, latrophilin and seven transmembrane domain containing 1 | NM_022159 |
| armadillo repeat gene deletes in velocardiofacial syndrome | BG149428 |
| microtubule-associated protein, RP/EB family, member 3 | AI885178 |
| espin | AL136880 |
| Similar to beta-1,4-mannosyltransferase; beta-1,4 mannosyltransferase | AK026807 |
| hypothetical protein FLJ33996 | R49644 |
| phosphatase, orphan 1 | AI016183 |
| Chromosome 6 open reading frame 204 | NM_012107 |
| glutaminyl-peptide cyclotransferase (glutaminyl cyclase) | NM_012413 |
| interferon, alpha-inducible protein (clone IFI-15K) | NM_005101 |
| Bone morphogenetic protein 8b (osteogenic protein 2) | AA610122 |
| chromosome 18 open reading frame 4 | AU119545 |
| Wolfram syndrome 1 (wolframin) | NM_006005 |
| hypothetical protein MGC14126 | AY099509 |
| dystroglycan 1 (dystrophin-associated glycoprotein 1) | AW411370 |
| hypothetical protein LOC283129 | AI688721 |
| chromosome 15 open reading frame 16 | NM_130901 |
| deleted in liver cancer 1 | AF026219 |
| Dystonin | BC020911 |
| coxsackie virus and adenovirus receptor | NM_001338 |
| macrophage scavenger receptor 1 | NM_002445 |
| mesenchymal stem cell protein DSC54 | NM_016644 |
| inhibitor of DNA binding 2, dominant negative helix-loop-helix protein /// inhibitor of DNA binding 2B, dominant negative helix-loop-helix protein | D13891 |
| hypothetical protein FLJ10159 | BF511724 |
| caspase 5, apoptosis-related cysteine protease | NM_004347 |
| chromosome 6 open reading frame 159 | AW511485 |
| Hypothetical protein FLJ23861 | AL706038 |
| arylacetamide deacetylase-like 1 | AB037784 |
| D4, zinc and double PHD fingers family 1 | NM_004647 |
| C1q and tumor necrosis factor related protein 2 | AF329836 |
| platelet-activating factor receptor | D10202 |
| gb:BF110321 /DB_XREF=gi:10940011 /DB_XREF=7n36e07.x1 /CLONE=IMAGE:3566700 /FEA=EST /CNT=8 /TID=Hs.117964.0 /TIER=ConsEnd /STK=4 /UG=Hs.117964 /UG_TITLE=ESTs | BF110321 |
| pleckstrin homology domain containing, family H (with MyTH4 domain) member 2 | AW451832 |
| Cdk5 and Abl enzyme substrate 2 | AI910855 |
| hypothetical protein FLJ40432 | AW135279 |
| PTEN induced putative kinase 1 | BE551215 |
| kallikrein 8 (neuropsin/ovasin) | NM_007196 |
| Hypothetical protein FLJ22662 | AA437213 |
| ATP-binding cassette, sub-family B (MDR/TAP), member 9 | NM_019625 |
| LOC440869 | AI806378 |
| glucuronyl C5-epimerase | W87398 |
| gb:AL035067 /DB_XREF=gi:4455454 /FEA=DNA /CNT=1 /TID=Hs.247844.0 /TIER=ConsEnd /STK=0 /UG=Hs.247844 /UG_TITLE=Human DNA sequence from clone 170F5 on chromosome Xq22.3-24. Contains an HMG1 (high-mobility group (nonhistone chromosomal) protein 1) pseudogene | AL035067 |
| Cbl-interacting protein Sts-1 | AI418293 |
| Full length insert cDNA clone ZD77F06 | AI655611 |
| RecQ protein-like 5 | AW028687 |
| KIAA1109 | AL137384 |
| 2'-5'-oligoadenylate synthetase-like | NM_003733 |
| tumor necrosis factor (ligand) superfamily, member 9 | NM_003811 |
| MRNA, Xq terminal portion. | D16471 |
| Transcribed locus, moderately similar to XP_497042.1 hypothetical gene supported by AK091508 [Homo sapiens] | AW340015 |
| cAMP responsive element binding protein 5 | AI819043 |
| chromosome 10 open reading frame 56 | AK024784 |
| G protein-coupled receptor 4 | NM_005282 |
| fascin homolog 2, actin-bundling protein, retinal (Strongylocentrotus purpuratus) | NM_012418 |
| hypothetical protein MGC12972 | BC005064 |
| gb:AI803010 /DB_XREF=gi:5368482 /DB_XREF=tj60c11.x1 /CLONE=IMAGE:2145908 /FEA=EST /CNT=7 /TID=Hs.126877.0 /TIER=ConsEnd /STK=0 /UG=Hs.126877 /UG_TITLE=ESTs | AI803010 |
| retinol binding protein 7, cellular | AI733027 |
| ATPase, Na+/K+ transporting, beta 3 polypeptide | AI928218 |
| C-type lectin domain family 7, member A /// C-type lectin domain family 7, member A | AF313468 |
| hypothetical protein DKFZp762H185 | H27948 |
| similar to metallo-beta-lactamase superfamily protein | BC038230 |
| ATP-binding cassette, sub-family B (MDR/TAP), member 9 | BE504895 |
| Neurexin 1 | AK093260 |
| nidogen (enactin) | BF940043 |
| cryptochrome 1 (photolyase-like) | D83702 |
| wingless-type MMTV integration site family, member 16 | AF169963 |
| signal-induced proliferation-associated 1 like 3 | AA425633 |
| mitogen-activated protein kinase 11 | NM_002751 |
| gb:BC043440.1 /DB_XREF=gi:27693403 /TID=Hs2.438464.1 /CNT=2 /FEA=mRNA /TIER=ConsEnd /STK=1 /UG=Hs.438464 /UG_TITLE=Homo sapiens, clone IMAGE:5297340, mRNA /DEF=Homo sapiens, clone IMAGE:5297340, mRNA. | BC043440 |
| histone 1, H2bc | NM_003526 |
| UL16 binding protein 2 | AA831769 |
| ankyrin repeat domain 25 | NM_015493 |
| suppressor of cytokine signaling 3 | AI244908 |
| suppressor of cytokine signaling 1 | AB005043 |
| protocadherin beta 17 pseudogene | AF152527 |
| Transcribed locus | AA002211 |
| cysteine and tyrosine-rich 1 | H06649 |
| gb:BF433885 /DB_XREF=gi:11446008 /DB_XREF=7q56a10.x1 /CLONE=IMAGE:3702067 /FEA=EST /CNT=5 /TID=Hs.128245.0 /TIER=ConsEnd /STK=5 /UG=Hs.128245 /UG_TITLE=ESTs | BF433885 |
| CDNA FLJ37332 fis, clone BRAMY2019710 | AI972016 |
| Cbp/p300-interacting transactivator, with Glu/Asp-rich carboxy-terminal domain, 2 | AF109161 |
| erythropoietin receptor | X97671 |
| chromosome 14 open reading frame 91 | AF113687 |
| Transcribed locus | BG392518 |
| Erbb2 interacting protein | BC038097 |
| Transcribed locus | BE892293 |
| nudix (nucleoside diphosphate linked moiety X)-type motif 12 | BC042967 |
| hypothetical protein FLJ31547 | NM_145024 |
| Sec1 family domain containing 1 | AA808051 |
| suppressor of cytokine signaling 3 | BG035761 |
| heat shock 70kDa protein 1A /// heat shock 70kDa protein 1B | NM_005345 |
| pyridoxal (pyridoxine, vitamin B6) phosphatase | BC000320 |
| dopamine receptor D4 | NM_000797 |
| von Willebrand factor | NM_000552 |
| hypothetical protein FLJ14721 | BE551088 |
| tissue inhibitor of metalloproteinase 3 (Sorsby fundus dystrophy, pseudoinflammatory) | U67195 |
| Ncaml | NM_016637 |
| Bobby sox homolog (Drosophila) | AI076370 |
| hairy/enhancer-of-split related with YRPW motif-like | NM_014571 |
| solute carrier family 2 (facilitated glucose transporter), member 3 /// solute carrier family 2 (facilitated glucose transporter), member 14 | AL110298 |
| nucleosome assembly protein 1-like 5 | AW025330 |
| Hypothetical protein FLJ20366 | H09269 |
| MARVEL domain containing 3 | BC005052 |
| solute carrier organic anion transporter family, member 4A1 | NM_016354 |
| Sec61 alpha 2 subunit (S. cerevisiae) | NM_018144 |
| Gephyrin | AU145365 |
| chromosome 7 open reading frame 13 | AF063598 |
| hypothetical protein FLJ22390 | NM_022746 |
| mucolipin 1 | NM_020533 |
| isocitrate dehydrogenase 3 (NAD+) alpha | AI826060 |
| Homo sapiens, clone IMAGE:4157625, mRNA | BG396868 |
| Transcribed locus, weakly similar to NP_061913.2 elongation protein 4 homolog (S. cerevisiae) [Homo sapiens] | H12214 |
| gb:U80774.1 /DB_XREF=gi:2231377 /FEA=mRNA /CNT=3 /TID=Hs.326800.0 /TIER=ConsEnd /STK=0 /UG=Hs.326800 /UG_TITLE=Human EST clone 53125 mariner transposon Hsmar1 sequence /DEF=Human EST clone 53125 mariner transposon Hsmar1 sequence. | U80774 |
| guanine nucleotide binding protein (G protein), alpha transducing activity polypeptide 1 | NM_000172 |
| TRK-fused gene | AI908188 |
| Hypothetical protein LOC284591 | AL079648 |
| synaptotagmin IX | BC029605 |
| RAP1 interacting factor homolog (yeast) | AU150841 |
| ankyrin 1, erythrocytic /// ankyrin 1, erythrocytic | M28880 |
| C-Maf-inducing protein | AF220234 |
| Transcribed locus, moderately similar to XP_517655.1 similar to KIAA0825 protein [Pan troglodytes] | BF114815 |
| plexin D1 | AB014520 |
| RNA binding protein with multiple splicing | AK025893 |
| NAD(P)H dehydrogenase, quinone 2 | NM_000904 |
| NGNL6975 | AK095550 |
| Full length insert cDNA clone YX74D05 | AI655467 |
| docking protein 5 | BC008992 |
| Transcribed locus | AW275011 |
| hypothetical protein LOC285986 | AI681644 |
| membrane-associated ring finger (C3HC4) 3 | AW593996 |
| zinc finger protein 165 | NM_003447 |
| Kelch-like 7 (Drosophila) | T57946 |
| hyaluronoglucosaminidase 1 | AF173154 |
| DEAD (Asp-Glu-Ala-Asp) box polypeptide 58 | NM_014314 |
| chromosome 20 open reading frame 128 | AK091138 |
| SH3-domain GRB2-like pseudogene 2 | X99660 |
| myo-inositol 1-phosphate synthase A1 | AL137749 |
| hypothetical protein MGC39681 | BC029583 |
| bone morphogenetic protein 2 | NM_001200 |
| Thyroid adenoma associated | AI674059 |
| gb:BF507344 /DB_XREF=gi:11590651 /DB_XREF=UI-H-BW1-amx-b-03-0-UI.s1 /CLONE=IMAGE:3071189 /FEA=EST /CNT=19 /TID=Hs.22301.0 /TIER=Stack /STK=9 /UG=Hs.22301 /UG_TITLE=ESTs | BF507344 |
| acid phosphatase-like 2 | AW069729 |
| LIM domain binding 2 | AU147267 |
| Deleted in a mouse model of primary ciliary dyskinesia | AW626574 |
| KIAA1276 protein | AL138198 |
| phospholipase C-like 2 | AK023546 |
| gb:BF244402 /DB_XREF=gi:11158333 /DB_XREF=601862985F1 /CLONE=IMAGE:4080500 /FEA=EST /CNT=6 /TID=Hs.97079.0 /TIER=ConsEnd /STK=0 /UG=Hs.97079 /UG_TITLE=ESTs | BF244402 |
| chromosome X open reading frame 1 | NM_004709 |
| Zinc finger protein 272 | AF113692 |
| PDZ and LIM domain 3 | BE840636 |
| reticulon 2 | NM_005619 |
| ASCL830 | AI871385 |
| hypothetical protein LOC286068 | AK096200 |
| potassium inwardly-rectifying channel, subfamily J, member 13 | AJ007557 |
| insulin-like growth factor binding protein 7 | AW770896 |
| KIAA0999 protein | BC035583 |
| Cyclic AMP-regulated phosphoprotein, 21 kD | AL049254 |
| ELAV (embryonic lethal, abnormal vision, Drosophila)-like 4 (Hu antigen D) | S73887 |
| LOC440173 | AI818652 |
| lemur tyrosine kinase 3 | BE868592 |
| hypothetical protein FLJ25169 | NM_152568 |
| Ly6/neurotoxin 1 | AV696976 |
| chromosome 2 open reading frame 31 /// chromosome 2 open reading frame 31 | NM_030804 |
| hypothetical protein FLJ13154 | BC006291 |
| Cullin 3 | AA960963 |
| Histone 1, H3j | AL522145 |
| Transcribed locus, weakly similar to XP_517454.1 similar to hypothetical protein MGC45438 [Pan troglodytes] | AI278445 |
| Intercellular adhesion molecule 3 | AI690006 |
| transient receptor potential cation channel, subfamily M, member 6 /// transient receptor potential cation channel, subfamily M, member 6 | AF350881 |
| transmembrane protein 46 | AW664964 |
| NOL1/NOP2/Sun domain family, member 3 | BC018432 |
| Hypothetical gene supported by BC034777 | BC034777 |
| SRY (sex determining region Y)-box 6 | AW300085 |
| solute carrier family 7 (cationic amino acid transporter, y+ system), member 7 | NM_003982 |
| peripheral myelin protein 22 | L03203 |
| C-type lectin domain family 7, member A | AF400600 |
| chemokine (C-X-C motif) ligand 14 | NM_004887 |
| hypothetical protein FLJ10052 | BC004888 |
| dystonin | AK025142 |
| pleckstrin homology domain containing, family B (evectins) member 2 | NM_017958 |
| gb:AI631993 /DB_XREF=gi:4683323 /DB_XREF=wa38e10.x1 /CLONE=IMAGE:2300394 /FEA=EST /CNT=26 /TID=Hs.145875.0 /TIER=Stack /STK=21 /UG=Hs.145875 /UG_TITLE=ESTs | AI631993 |
| a disintegrin and metalloproteinase domain 8 /// a disintegrin and metalloproteinase domain 8 | NM_001109 |
| Chloride channel 4 | W26966 |
| gb:D88435.1 /DB_XREF=gi:2506079 /FEA=FLmRNA /CNT=153 /TID=Hs.153227.0 /TIER=ConsEnd /STK=0 /UG=Hs.153227 /LL=2580 /UG_GENE=GAK /DEF=Homo sapiens mRNA for HsGAK, complete cds. /PROD=HsGAK /FL=gb:NM_005255.1 gb:D88435.1 | D88435 |
| carbohydrate (chondroitin 4) sulfotransferase 12 | NM_018641 |
| histone 1, H2ba | AI932318 |
| Niemann-Pick disease, type C1 | NM_000271 |
| serine (or cysteine) proteinase inhibitor, clade B (ovalbumin), member 4 /// serine (or cysteine) proteinase inhibitor, clade B (ovalbumin), member 4 | AB046400 |
| Thy-1 cell surface antigen /// Thy-1 co-transcribed | AL161958 |
| stomatin | M81635 |
| potassium voltage-gated channel, KQT-like subfamily, member 1 | U89364 |
| brain expressed X-linked-like 1 | AL523320 |
| BAI1-associated protein 2 | AB017120 |
| septin 4 | U88870 |
| hypothetical protein DKFZp434K1815 | BE896303 |
| hypothetical protein LOC145474 | AU158212 |
| dual specificity phosphatase 1 | NM_004417 |
| similar to cDNA sequence BC035954 | BE676352 |
| solute carrier family 4, sodium bicarbonate cotransporter, member 7 | AF053755 |
| Elongation factor RNA polymerase II | AL050168 |
| abl-interactor 1 | BF194851 |
| Tyrosine kinase with immunoglobulin-like and EGF-like domains 1 | AL833389 |
| Yippee-like 2 (Drosophila) | AW005748 |
| V-maf musculoaponeurotic fibrosarcoma oncogene homolog (avian) | BE674528 |
| CDNA FLJ39484 fis, clone PROST2014925 | AK057259 |
| G protein-coupled receptor kinase 5 | NM_005308 |
| S100 calcium binding protein A7 (psoriasin 1) | NM_002963 |
| deiodinase, iodothyronine, type III opposite strand | AI560757 |
| ASF1 anti-silencing function 1 homolog B (S. cerevisiae) | NM_018154 |
| sema domain, transmembrane domain (TM), and cytoplasmic domain, (semaphorin) 6B | AB022433 |
| hypothetical protein LOC200008 | BF438410 |
| gb:AW190479 /DB_XREF=gi:6464959 /DB_XREF=xl15g03.x1 /CLONE=IMAGE:2676340 /FEA=EST /CNT=7 /TID=Hs.177948.0 /TIER=ConsEnd /STK=4 /UG=Hs.177948 /UG_TITLE=ESTs, Weakly similar to ALU5_HUMAN ALU SUBFAMILY SC SEQUENCE CONTAMINATION WARNING ENTRY (H.sapiens) | AW190479 |
| potassium channel, subfamily K, member 7 | AF110523 |
| tumor necrosis factor, alpha-induced protein 2 | NM_006291 |
| density-regulated protein | AW665791 |
| SAM domain and HD domain 1 | NM_015474 |
| Lymphocyte antigen 6 complex, locus K | AI828018 |
| G protein-coupled receptor 155 | AI733474 |
| 6-phosphofructo-2-kinase/fructose-2,6-biphosphatase 3 | NM_004566 |
| Transcribed locus | AI421677 |
| hypothetical protein FLJ31713 | NM_152575 |
| zinc finger protein 143 (clone pHZ-1) | NM_003442 |
| zinc finger protein 407 | NM_017757 |
| PDZ domain containing 1 | AI916532 |
| sialidase 1 (lysosomal sialidase) | U84246 |
| purine-rich element binding protein G | NM_013357 |
| nuclear factor (erythroid-derived 2)-like 3 | NM_004289 |
| gb:AL034410 /DB_XREF=gi:4678510 /FEA=DNA /CNT=1 /TID=Hs.247846.0 /TIER=ConsEnd /STK=0 /UG=Hs.247846 /UG_TITLE=Human DNA sequence from clone 774G10 on chromosome Xp11.23-11.3 Contains a pseudogene similar to cyclin protein, ESTs, STS and GSSs /DEF=Human DN | AL034410 |
| gb:BC043438.1 /DB_XREF=gi:27693846 /TID=Hs2.438446.1 /CNT=2 /FEA=mRNA /TIER=ConsEnd /STK=1 /UG=Hs.438446 /UG_TITLE=Homo sapiens, clone IMAGE:5297032, mRNA /DEF=Homo sapiens, clone IMAGE:5297032, mRNA. | BC043438 |
| fibronectin type III domain containing 3A | BE675600 |
| carboxypeptidase M | NM_001874 |
| spermatogenesis associated 11 | AA774555 |
| zinc finger, CCHC domain containing 14 | AL117532 |
| gb:BC043266.1 /DB_XREF=gi:27693378 /TID=Hs2.438469.1 /CNT=2 /FEA=mRNA /TIER=ConsEnd /STK=1 /UG=Hs.438469 /UG_TITLE=Homo sapiens, clone IMAGE:5296922, mRNA /DEF=Homo sapiens, clone IMAGE:5296922, mRNA. | BC043266 |
| zinc finger protein 179 | AB026054 |
| claudin 14 | AF314090 |
| frizzled homolog 4 (Drosophila) /// frizzled homolog 4 (Drosophila) | AB054881 |
| fucosyltransferase 4 (alpha (1,3) fucosyltransferase, myeloid-specific) | M58596 |
| apoptosis-inducing factor (AIF)-like mitochondrion-associated inducer of death | AI922797 |
| topoisomerase (DNA) I | AW025108 |
| G protein-coupled receptor 22 | NM_005295 |
| Hypothetical protein DKFZp547K1113 | AL109698 |
| CDNA FLJ37302 fis, clone BRAMY2016009 | AW518929 |
| glutamate receptor, ionotropic, N-methyl-D-aspartate 3A | AL359651 |
| Full-length cDNA clone CS0DK008YI09 of HeLa cells Cot 25-normalized of Homo sapiens (human) | AI357639 |
| keratin 19 pseudogene | AB041269 |
| hypothetical protein FLJ20255 | NM_017728 |
| islet cell autoantigen 1, 69kDa /// islet cell autoantigen 1, 69kDa | BC005922 |
| synovial sarcoma, X breakpoint 3 | NM_021014 |
| gb:AW972850 /DB_XREF=gi:8162696 /DB_XREF=EST384945 /FEA=EST /CNT=9 /TID=Hs.294083.1 /TIER=ConsEnd /STK=0 /UG=Hs.294083 /UG_TITLE=ESTs | AW972850 |
| Transcribed locus | AA007596 |
| MAS-related GPR, member F | H15920 |
| MYC associated factor X | AI346181 |
| glycoprotein (transmembrane) nmb | BC011595 |
| gb:BC028592.1 /DB_XREF=gi:20306901 /TID=Hs2.367948.1 /CNT=2 /FEA=FLmRNA /TIER=FL /STK=1 /UG=Hs.367948 /DEF=Homo sapiens, Similar to Another transcription unit, clone MGC:27180 IMAGE:4289497, mRNA, complete cds. /PROD=Similar to Another transcription unit | BC028592 |
| Chondroitin sulfate GalNAcT-2 | BE009751 |
| silver homolog (mouse) | U01874 |
| Eukaryotic translation initiation factor 4E member 3 | AL832401 |
| Succinate dehydrogenase complex, subunit A, flavoprotein-like 2 | AW090199 |
| Full-length cDNA clone CS0DF012YG01 of Fetal brain of Homo sapiens (human) | BG260087 |
| EST from clone 208499, full insert | AL355688 |
| transmembrane protein 35 | NM_021637 |
| glutaryl-Coenzyme A dehydrogenase | NM_000159 |
| Homo sapiens, clone IMAGE:5297356, mRNA | AW665239 |
| hydroxysteroid (17-beta) dehydrogenase 6 | U89281 |
| Acyl-CoA synthetase long-chain family member 3 | AL525798 |
| chromosome 11 open reading frame 9 | AC004770 |
| phosphodiesterase 4A, cAMP-specific (phosphodiesterase E2 dunce homolog, Drosophila) | NM_006202 |
| Transcribed locus | AA971931 |
| hypothetical protein DKFZp434N035 | AL136879 |
| Transcribed locus | AW274369 |
| gb:AI039329 /DB_XREF=gi:3278523 /DB_XREF=ox36c12.s1 /CLONE=IMAGE:1658422 /FEA=EST /CNT=6 /TID=Hs.124058.0 /TIER=ConsEnd /STK=5 /UG=Hs.124058 /UG_TITLE=ESTs | AI039329 |
| Ankyrin repeat and sterile alpha motif domain containing 1 | AI583964 |
| HANP1 | AI807042 |
| transition protein 2 (during histone to protamine replacement) | X63759 |
| myeloma overexpressed gene (in a subset of t(11;14) positive multiple myelomas) | AA621983 |
| adaptor-related protein complex 2, alpha 1 subunit | AC006942 |
| interferon regulatory factor 7 | NM_004030 |
| CDNA FLJ26164 fis, clone ADG02723 | BE501087 |
| KIAA0574 protein | AL512740 |
| Kelch repeat and BTB (POZ) domain containing 7 | BE672291 |
| septin 3 | AF285109 |
| pecanex-like 2 (Drosophila) | NM_024938 |
| SEC15-like 1 (S. cerevisiae) | AK002113 |
| Endothelin converting enzyme 2 | AI084921 |
| Presenilin 1 (Alzheimer disease 3) | AI659439 |
| isocitrate dehydrogenase 3 (NAD+) alpha | NM_005530 |
| solute carrier family 1 (neuronal/epithelial high affinity glutamate transporter, system Xag), member 1 | AW235061 |
| Transcribed locus | BE960968 |
| Transcribed locus | H24302 |
| hypothetical protein MGC32805 | BC029465 |
| PTPRF interacting protein, binding protein 2 (liprin beta 2) | AI692180 |
| allograft inflammatory factor 1 | AF299327 |
| gb:AF119882.1 /DB_XREF=gi:7770200 /FEA=FLmRNA /CNT=2 /TID=Hs.283039.0 /TIER=FL /STK=0 /UG=Hs.283039 /LL=55398 /UG_GENE=PRO2492 /DEF=Homo sapiens PRO2492 mRNA, complete cds. /PROD=PRO2492 /FL=gb:AF119882.1 | AF119882 |
| LIM and senescent cell antigen-like domains 3 | AF288404 |
| Odz, odd Oz/ten-m homolog 3 (Drosophila) | AI928518 |
| Transcribed locus | BF511339 |
| WD repeat domain 17 | BI713506 |
| hypothetical protein LOC283674 | AK092120 |
| MRNA; cDNA DKFZp564C163 (from clone DKFZp564C163) | AL049244 |
| UDP-glucose ceramide glucosyltransferase | NM_003358 |
| similar to Argininosuccinate synthase (Citrulline--aspartate ligase) | AC003989 |
| Transcribed locus | W86478 |
| solute carrier family 29 (nucleoside transporters), member 3 | NM_018344 |
| protein kinase NYD-SP25 | AI041556 |
| Protein tyrosine phosphatase, receptor type, f polypeptide (PTPRF), interacting protein (liprin), alpha 2 | AA885753 |
| cyclin M4 | NM_020184 |
| CD47 antigen (Rh-related antigen, integrin-associated signal transducer) | BG230614 |
| phosphatidylinositol-4-phosphate 5-kinase, type I, gamma | AB011161 |
| gb:L29536.1 /DB_XREF=gi:463081 /GEN=CACNL1A1 /FEA=FLmRNA /CNT=1 /TID=Hs.89925.2 /TIER=FL /STK=0 /UG=Hs.89925 /LL=775 /DEF=Human calcium channel L-type alpha 1 subunit (CACNL1A1) mRNA, complete cds. /PROD=calcium channel L-type alpha 1 subunit /FL=gb:L2953 | L29536 |
| SH3 domain containing, Ysc84-like 1 (S. cerevisiae) | AW811976 |
| Full length insert cDNA clone YY74E10 | AF088010 |
| Transcribed locus, strongly similar to NP_536852.1 Homo sapiens ND4 gene | AW022267 |
| hypothetical protein FLJ32214 | NM_152473 |
| nitric oxide synthase 3 antisense | NM_173681 |
| glycerophosphodiester phosphodiesterase domain containing 1 | AK094770 |
| zinc finger protein 236 | NM_007345 |
| chemokine (C-X-C motif) ligand 14 | AF144103 |
| Matrilin 3 | AI809899 |
| CDNA: FLJ20908 fis, clone ADSE00417 | AK024561 |
| hypothetical LOC389440 | AA776715 |
| 5-azacytidine induced 1 | AB029041 |
| chromosome 6 open reading frame 174 | AL096711 |
| hypothetical protein LOC283761 | BC039350 |
| gb:BF110669 /DB_XREF=gi:10940359 /DB_XREF=7n55h03.x1 /CLONE=IMAGE:3568733 /FEA=EST /CNT=22 /TID=Hs.143408.0 /TIER=Stack /STK=15 /UG=Hs.143408 /UG_TITLE=ESTs | BF110669 |
| Chemokine-like factor super family 6 | AW301108 |
| PR domain containing 2, with ZNF domain | U23736 |
| Hypothetical protein LOC283501 | U90905 |
| myosin VA (heavy polypeptide 12, myoxin) | Z22957 |
| Transcribed locus, moderately similar to NP_055301.1 neuronal thread protein AD7c-NTP [Homo sapiens] | BE670451 |
| KIAA1702 protein | BC035337 |
| CDNA clone IMAGE:3456494, partial cds | BG178274 |
| phenylalanine hydroxylase | H47984 |
| ATPase, H+ transporting, lysosomal V0 subunit a isoform 1 | AL096733 |
| secretory carrier membrane protein 5 | BE222801 |
| orosomucoid 1 | NM_000607 |
| Ras-related associated with diabetes | AA031330 |
| PDZ domain containing, X chromosome | BG413572 |
| GRB2-associated binding protein 2 | NM_012296 |
| capping protein (actin filament) muscle Z-line, alpha 3 | AI654717 |
| transforming, acidic coiled-coil containing protein 1 | NM_006283 |
| oxidised low density lipoprotein (lectin-like) receptor 1 | AF035776 |
| SRY (sex determining region Y)-box 10 | BC002824 |
| adenylate kinase 1 | NM_000476 |
| gb:BC041892.1 /DB_XREF=gi:27469434 /TID=Hs2.434544.1 /CNT=1 /FEA=mRNA /TIER=ConsEnd /STK=1 /UG=Hs.434544 /UG_TITLE=Homo sapiens, clone IMAGE:5298624, mRNA /DEF=Homo sapiens, clone IMAGE:5298624, mRNA. | BC041892 |
| keratin, hair, acidic, 2 | NM_002278 |
| kelch-like 15 (Drosophila) | BG149487 |
| gb:AK024445.1 /DB_XREF=gi:10440403 /GEN=FLJ00035 /FEA=mRNA /CNT=2 /TID=Hs.287752.0 /TIER=ConsEnd /STK=0 /UG=Hs.287752 /DEF=Homo sapiens mRNA for FLJ00035 protein, partial cds. /PROD=FLJ00035 protein | AK024445 |
| chromodomain helicase DNA binding protein 7 | NM_017783 |
| ribosomal protein S6 kinase, 90kDa, polypeptide 2 | AI992251 |
| suppressor of variegation 4-20 homolog 2 (Drosophila) | AF289582 |
| ring finger protein 24 | NM_007219 |
| guanine nucleotide binding protein (G protein), alpha 11 (Gq class) | NM_002067 |
| CDNA clone IMAGE:6025865, partial cds | AA156240 |
| leucine rich repeat neuronal 5 | AK024867 |
| gb:AA625872 /DB_XREF=gi:2538259 /DB_XREF=zu92c10.s1 /CLONE=IMAGE:745458 /FEA=EST /CNT=4 /TID=Hs.98977.0 /TIER=ConsEnd /STK=4 /UG=Hs.98977 /UG_TITLE=ESTs, Moderately similar to T34561 hypothetical protein DKFZp434L1050.1 (H.sapiens) | AA625872 |
| Ubiquitin-conjugating enzyme E2S | BC031290 |
| protein phosphatase 1, regulatory subunit 10 | AI492873 |
| dihydrodiol dehydrogenase (dimeric) | NM_014475 |
| BAI1-associated protein 2 | NM_017450 |
| hedgehog interacting protein | AW444502 |
| MRNA; cDNA DKFZp667A1719 (from clone DKFZp667A1719) | AL832295 |
| HGF activator | NM_001528 |
| acyl-CoA synthetase long-chain family member 3 | D89053 |
| chromosome 1 open reading frame 36 | AA719822 |
| gb:X81445 /DB_XREF=gi:7671636 /FEA=DNA /CNT=1 /TID=Hs.278905.0 /TIER=ConsEnd /STK=0 /UG=Hs.278905 /LL=26537 /UG_GENE=OR51A1P /UG_TITLE=olfactory receptor, family 51, subfamily A, member 1 pseudogene /DEF=Homo spaiens HPFH6OR gene for olfactory receptor | X81445 |
| carboxypeptidase, vitellogenic-like /// carboxypeptidase, vitellogenic-like | NM_031311 |
| Homo sapiens, clone IMAGE:5222445, mRNA | BC041470 |
| similar to aspartate beta hydroxylase (ASPH) | Z99714 |
| LOC441301 | AI949265 |
| phosphatidylinositol transfer protein, alpha | H15647 |
| gb:BF792773 /DB_XREF=gi:12097827 /DB_XREF=602253318F1 /CLONE=IMAGE:4345660 /FEA=EST /CNT=7 /TID=Hs.144900.0 /TIER=ConsEnd /STK=3 /UG=Hs.144900 /UG_TITLE=ESTs | BF792773 |
| A kinase (PRKA) anchor protein (yotiao) 9 | AK000270 |
| isocitrate dehydrogenase 2 (NADP+), mitochondrial | AU151428 |
| CDNA clone IMAGE:5302913, partial cds | AI631888 |
| Ubiquitin protein ligase E3 component n-recognin 2 | AK001118 |
| SAM domain and HD domain 1 | AF147427 |
| transmembrane protein 47 | AL136550 |
| ORM1-like 3 (S. cerevisiae) | BF337528 |
| Dual adaptor of phosphotyrosine and 3-phosphoinositides | AW297879 |
| Transcribed locus, strongly similar to NP_077136.2 mitochondrial ribosomal protein S23 [Mus musculus] | AI274352 |
| hypothetical protein LOC286411 | AV753446 |
| gb:AC007292 /DB_XREF=gi:4581080 /FEA=DNA_3 /CNT=2 /TID=Hs.76925.1 /TIER=ConsEnd /STK=0 /UG=Hs.76925 /UG_TITLE=Homo sapiens chromosome 19, cosmid R31167 /DEF=Homo sapiens chromosome 19, cosmid R31167 | AC007292 |
| Heterogeneous nuclear ribonucleoprotein A/B | AI760020 |
| Calsyntenin 3 | AW449674 |
| Hypothetical protein LOC284669 | AK092251 |
