# Supplementary material for: MAGEB2 is Activated by Promoter Demethylation in Head and Neck Squamous Cell Carcinoma
Source: PLoS One. 2012 Sep 24;7(9):e45534. doi: 10.1371/journal.pone.0045534 (PMC3454438; doi:10.1371/journal.pone.0045534)
Supplement: Table S4 — Top ranked 178 genes. (DOCX) [file pone.0045534.s008.docx]

**Supplementary Table 4- Top ranked 178 genes**

| Gene Name | Accession # | |
| --- | --- | --- |
| melanoma antigen family A, 4 | | AW438674 |
| melanoma antigen family A, 6 | | U10691 |
| melanoma antigen family A, 3 | | BC000340 |
| homeo box HB9 | | AI738662 |
| melanoma antigen family A, 12 | | BC003408 |
| cytokeratin type II | | NM_004693 |
| heat shock 70kDa protein 2 | | U56725 |
| solute carrier family 8 (sodium-calcium exchanger), member 2 | | AI127885 |
| melanoma antigen family A, 11 | | BC004479 |
| melanoma antigen family B, 2 | | NM_002364 |
| astrotactin 2 | | AF116574 |
| cysteine-rich secretory protein LCCL domain containing 2 | | AL136861 |
| apolipoprotein C-I | | NM_001645 |
| contactin associated protein-like 2 | | AC005378 |
| transketolase-like 1 | | Z49258 |
| distal-less homeo box 2 | | NM_004405 |
| ornithine decarboxylase 1 | | NM_002539 |
| keratin, hair, basic, 6 (monilethrix) | | X99142 |
| chromosome 6 open reading frame 148 | | NM_030568 |
| ubiquitin carboxyl-terminal esterase L1 (ubiquitin thiolesterase) | | NM_004181 |
| oxoglutarate dehydrogenase-like | | NM_018245 |
| collagen, type IX, alpha 3 | | NM_001853 |
| collagen triple helix repeat containing 1 | | AA584310 |
| DnaJ (Hsp40) homolog, subfamily C, member 6 | | AV729634 |
| KIPV467 | | W69083 |
| paternally expressed 10 | | AL582836 |
| tripartite motif-containing 9 | | AF220036 |
| hypothetical protein FLJ90166 | | BG326592 |
| transmembrane protein SHREW1 | | NM_018836 |
| fibulin 2 | | NM_001998 |
| keratin, hair, basic, 1 | | NM_002281 |
| RAB3B, member RAS oncogene family | | BC005035 |
| Hypothetical protein MGC42174 | | AL832765 |
| cysteine and glycine-rich protein 2 | | U46006 |
| dynamin 3 | | AI631915 |
| protein tyrosine phosphatase, receptor type, f polypeptide (PTPRF), interacting protein (liprin), alpha 1 | | U22815 |
| chondroitin sulfate proteoglycan 5 (neuroglycan C) | | AF059274 |
| Solute carrier family 7 (cationic amino acid transporter, y+ system), member 8 | | AL365343 |
| gb:BF111214 /DB_XREF=gi:10940904 /DB_XREF=7n44e07.x1 /CLONE=IMAGE:3567468 /FEA=EST /CNT=5 /TID=Hs.128138.0 /TIER=ConsEnd /STK=5 /UG=Hs.128138 /UG_TITLE=ESTs, Weakly similar to ATS1_HUMAN ADAM-TS 1 PRECURSOR (H.sapiens) | | BF111214 |
| carnitine palmitoyltransferase 1C | | AL565745 |
| protein phosphatase 1, regulatory (inhibitor) subunit 14A | | AA156998 |
| dipeptidylpeptidase 4 (CD26, adenosine deaminase complexing protein 2) | | M80536 |
| G protein-coupled receptor 54 | | AI819198 |
| hairy/enhancer-of-split related with YRPW motif 2 | | AF232238 |
| glutamate receptor, ionotropic, N-methyl D-aspartate 1 | | NM_007327 |
| dispatched homolog 2 (Drosophila) | | AB051529 |
| Ras-induced senescence 1 | | BF062629 |
| disabled homolog 1 (Drosophila) | | NM_021080 |
| kinesin family member 26A | | AK026406 |
| RAS, dexamethasone-induced 1 | | AF069506 |
| cadherin, EGF LAG seven-pass G-type receptor 3 (flamingo homolog, Drosophila) | | NM_001407 |
| KIAA1937 protein | | AK093300 |
| transcription factor AP-2 beta (activating enhancer binding protein 2 beta) | | NM_003221 |
| arachidonate 15-lipoxygenase, second type | | NM_001141 |
| Guanylate binding protein 5 | | BG545653 |
| insulin-like growth factor binding protein 3 | | BF340228 |
| SRY (sex determining region Y)-box 30 | | NM_007017 |
| chemokine (C-X-C motif) ligand 3 | | NM_002090 |
| transglutaminase 2 (C polypeptide, protein-glutamine-gamma-glutamyltransferase) | | M98478 |
| ras homolog gene family, member B | | AI263909 |
| RASD family, member 2 | | AF279143 |
| collagen, type II, alpha 1 (primary osteoarthritis, spondyloepiphyseal dysplasia, congenital) | | X16468 |
| myosin, light polypeptide kinase /// myosin, light polypeptide kinase | | NM_005965 |
| collagen, type V, alpha 3 | | NM_015719 |
| Suppression of tumorigenicity 7 like | | BF961733 |
| Exostoses (multiple) 1 | | BC017944 |
| intercellular adhesion molecule 1 (CD54), human rhinovirus receptor | | AI608725 |
| nanos homolog 1 (Drosophila) | | AW970089 |
| crystallin, alpha B | | AF007162 |
| peptidylprolyl isomerase (cyclophilin)-like 2 | | NM_014337 |
| DEAD (Asp-Glu-Ala-Asp) box polypeptide 43 | | NM_018665 |
| interleukin 20 | | AF224266 |
| Kell blood group precursor (McLeod phenotype) | | NM_021083 |
| DNA (cytosine-5-)-methyltransferase 3 beta | | NM_006892 |
| protocadherin 10 | | AI640307 |
| Similar to hypothetical protein FLJ20296 | | AK098125 |
| potassium channel, subfamily K, member 15 | | NM_022358 |
| endothelin 1 | | BC036851 |
| CDNA FLJ11397 fis, clone HEMBA1000622 | | AU144005 |
| relaxin 3 | | AB076563 |
| neuronal pentraxin II | | U26662 |
| TSC22 domain family 2 | | AF201291 |
| neurexophilin 4 | | AI933199 |
| sodium channel, voltage-gated, type I, beta | | NM_001037 |
| v-maf musculoaponeurotic fibrosarcoma oncogene homolog B (avian) | | NM_005461 |
| Transmembrane protein 41B | | N64760 |
| transglutaminase 2 (C polypeptide, protein-glutamine-gamma-glutamyltransferase) | | AL031651 |
| SMAD, mothers against DPP homolog 6 (Drosophila) | | NM_005585 |
| pentraxin-related gene, rapidly induced by IL-1 beta | | NM_002852 |
| Multiple C2-domains with two transmembrane regions 1 | | BG250585 |
| chemokine (C-X-C motif) ligand 11 | | AF030514 |
| Full open reading frame cDNA clone RZPDo834C0824D for gene HIST2H4, histone 2, H4; complete cds, incl. stopcodon | | AI828075 |
| dual specificity phosphatase 9 | | NM_001395 |
| dipeptidylpeptidase 4 (CD26, adenosine deaminase complexing protein 2) | | M74777 |
| doublesex and mab-3 related transcription factor 1 | | NM_021951 |
| hypothetical protein FLJ14351 | | NM_024732 |
| forkhead box protein O6 | | AI341823 |
| chromosome 9 open reading frame 140 | | AW250904 |
| cytochrome c oxidase subunit 8C | | AW269746 |
| cytochrome P450, family 24, subfamily A, polypeptide 1 | | NM_000782 |
| family with sequence similarity 20, member A | | AW291369 |
| prostaglandin-endoperoxide synthase 1 (prostaglandin G/H synthase and cyclooxygenase) | | S36219 |
| fatty acid 2-hydroxylase | | NM_024306 |
| lysosomal-associated membrane protein 3 | | NM_014398 |
| HORMA domain containing 1 | | AL136755 |
| synaptophysin | | U93305 |
| ATP-binding cassette, sub-family A (ABC1), member 4 | | U88667 |
| ADAMTS-like 2 | | NM_014694 |
| histone 1, H2bd | | AL353759 |
| hippocalcin-like 1 | | NM_002149 |
| reticulocalbin 3, EF-hand calcium binding domain | | AI797684 |
| carboxypeptidase A4 | | NM_016352 |
| small nuclear RNA activating complex, polypeptide 1, 43kDa | | NM_003082 |
| prostaglandin-endoperoxide synthase 1 (prostaglandin G/H synthase and cyclooxygenase) | | NM_000962 |
| synaptogyrin 3 | | NM_004209 |
| prostaglandin-endoperoxide synthase 1 (prostaglandin G/H synthase and cyclooxygenase) | | BE613133 |
| epiplakin 1 | | AL137725 |
| epiplakin 1 | | AL137725 |
| paternally expressed 10 | | BE858180 |
| transmembrane protein SHREW1 | | AA835004 |
| Solute carrier family 7 (cationic amino acid transporter, y+ system), member 8 | | AL365343 |
| cysteine and glycine-rich protein 2 | | NM_001321 |
| insulin-like growth factor binding protein 3 | | M31159 |
| ras homolog gene family, member B | | BI668074 |
| gb:R41431 /DB_XREF=gi:816734 /DB_XREF=yf95c10.s1 /CLONE=IMAGE:30249 /FEA=EST /CNT=4 /TID=Hs.22495.0 /TIER=ConsEnd /STK=4 /UG=Hs.22495 /UG_TITLE=ESTs | | R41431 |
| gb:AI394574 /DB_XREF=gi:4224121 /DB_XREF=tg08c12.x1 /CLONE=IMAGE:2108182 /FEA=EST /CNT=8 /TID=Hs.157123.0 /TIER=Stack /STK=8 /UG=Hs.157123 /UG_TITLE=ESTs | | AI394574 |
| dehydrogenase/reductase (SDR family) member 2 | | AK000345 |
| matrix metalloproteinase 13 (collagenase 3) /// matrix metalloproteinase 13 (collagenase 3) | | NM_002427 |
| hypothetical protein LOC254848 | | BC043614 |
| fatty acid binding protein 4, adipocyte | | NM_001442 |
| FYVE, RhoGEF and PH domain containing 2 | | AW516510 |
| plasminogen activator, tissue | | NM_000930 |
| dehydrogenase/reductase (SDR family) member 2 | | NM_005794 |
| RAB3B, member RAS oncogene family | | AU156710 |
| Transcribed locus, moderately similar to NP_055301.1 neuronal thread protein AD7c-NTP [Homo sapiens] | | AA922273 |
| Hypothetical protein MGC39606 | | AA565509 |
| CDNA clone IMAGE:5301169, partial cds | | BM479034 |
| insulin growth factor-like family member 1 | | AA195677 |
| retinol dehydrogenase 12 (all-trans and 9-cis) | | AI796235 |
| prion protein 2 (dublet) | | AL133396 |
| LOC440570 | | AI733297 |
| trophoblast-derived noncoding RNA | | AV659198 |
| interleukin 8 | | AF043337 |
| interleukin 1 receptor-like 1 | | NM_003856 |
| Transcribed locus | | AA876179 |
| hypothetical gene supported by AK123741 | | BF109906 |
| interleukin 8 | | NM_000584 |
| Transcribed locus | | BE670161 |
| hypothetical gene supported by AK125122 | | AA129774 |
| placenta-specific 1 | | NM_021796 |
| Full-length cDNA clone CS0DJ013YP06 of T cells (Jurkat cell line) Cot 10-normalized of Homo sapiens (human) | | H49805 |
| chemokine (C-X-C motif) ligand 11 | | AF002985 |
| similar to mouse 1700027M21Rik gene | | AI964053 |
| interleukin 6 (interferon, beta 2) | | NM_000600 |
| gb:AI810266 /DB_XREF=gi:5396832 /DB_XREF=wb86h07.x1 /CLONE=IMAGE:2312605 /FEA=EST /CNT=12 /TID=Hs.130853.0 /TIER=ConsEnd /STK=5 /UG=Hs.130853 /UG_TITLE=ESTs | | AI810266 |
| SPANX family, member B1 /// SPANX family, member B2 | | NM_013453 |
| Homo sapiens, clone IMAGE:3891572, mRNA | | BC009533 |
| trophoblast-derived noncoding RNA | | AU155361 |
| Homo sapiens, clone IMAGE:4822964, mRNA | | BC034800 |
| Transcribed locus, moderately similar to XP_517454.1 similar to hypothetical protein MGC45438 [Pan troglodytes] | | BF056092 |
| bone marrow stromal cell antigen 2 | | NM_004335 |
| collagen, type VIII, alpha 2 | | AI806793 |
| Xg blood group (pseudoautosomal boundary-divided on the X chromosome) | | AF380356 |
| Stanniocalcin 1 | | AW003173 |
| metastasis associated lung adenocarcinoma transcript 1 (non-coding RNA) | | AF132202 |
| keratin, hair, acidic, 4 | | NM_021013 |
| G protein-coupled receptor 24 | | AI934819 |
| S100 calcium binding protein A7-like 1 | | AJ243672 |
| small proline-rich protein 2G | | AA456642 |
| neuronal guanine nucleotide exchange factor | | AV703769 |
| unc-93 homolog A (C. elegans) | | AL021331 |
| lumican | | NM_002345 |
| MRNA; cDNA DKFZp686H1890 (from clone DKFZp686H1890) | | AI083557 |
| coagulation factor II (thrombin) receptor-like 2 | | AI378647 |
| solute carrier family 7, (cationic amino acid transporter, y+ system) member 13 | | AI471866 |
| defensin, beta 1 | | U73945 |
| protein tyrosine phosphatase, receptor type, f polypeptide (PTPRF), interacting protein (liprin), alpha 4 | | AK023365 |
| PDZ domain containing 1 | | NM_002614 |
|  | |  |
