# Supplementary material for: MAGEB2 is Activated by Promoter Demethylation in Head and Neck Squamous Cell Carcinoma
Source: PLoS One. 2012 Sep 24;7(9):e45534. doi: 10.1371/journal.pone.0045534 (PMC3454438; doi:10.1371/journal.pone.0045534)
Supplement: Table S5 — Top 96 genes. (DOCX) [file pone.0045534.s009.docx]

**Supplementary Table 5- Top 96 genes**

| Gene Name | Accession # |
| --- | --- |
| homeo box HB9 | AI738662 |
| heat shock 70kDa protein 2 | U56725 |
| solute carrier family 8 (sodium-calcium exchanger), member 2 | AI127885 |
| melanoma antigen family B, 2 | NM_002364 |
| astrotactin 2 | AF116574 |
| cysteine-rich secretory protein LCCL domain containing 2 | AL136861 |
| apolipoprotein C-I | NM_001645 |
| contactin associated protein-like 2 | AC005378 |
| distal-less homeo box 2 | NM_004405 |
| keratin, hair, basic, 6 (monilethrix) | X99142 |
| chromosome 6 open reading frame 148 | NM_030568 |
| oxoglutarate dehydrogenase-like | NM_018245 |
| collagen, type IX, alpha 3 | NM_001853 |
| collagen triple helix repeat containing 1 | AA584310 |
| DnaJ (Hsp40) homolog, subfamily C, member 6 | AV729634 |
| KIPV467 | W69083 |
| paternally expressed 10 | AL582836 |
| tripartite motif-containing 9 | AF220036 |
| hypothetical protein FLJ90166 | BG326592 |
| transmembrane protein SHREW1 | NM_018836 |
| fibulin 2 | NM_001998 |
| keratin, hair, basic, 1 | NM_002281 |
| RAB3B, member RAS oncogene family | BC005035 |
| Hypothetical protein MGC42174 | AL832765 |
| cysteine and glycine-rich protein 2 | U46006 |
| dynamin 3 | AI631915 |
| protein tyrosine phosphatase, receptor type, f polypeptide (PTPRF), interacting protein (liprin), alpha 1 | U22815 |
| chondroitin sulfate proteoglycan 5 (neuroglycan C) | AF059274 |
| Solute carrier family 7 (cationic amino acid transporter, y+ system), member 8 | AL365343 |
| gb:BF111214 /DB_XREF=gi:10940904 /DB_XREF=7n44e07.x1 /CLONE=IMAGE:3567468 /FEA=EST /CNT=5 /TID=Hs.128138.0 /TIER=ConsEnd /STK=5 /UG=Hs.128138 /UG_TITLE=ESTs, Weakly similar to ATS1_HUMAN ADAM-TS 1 PRECURSOR (H.sapiens) | BF111214 |
| protein phosphatase 1, regulatory (inhibitor) subunit 14A | AA156998 |
| G protein-coupled receptor 54 | AI819198 |
| hairy/enhancer-of-split related with YRPW motif 2 | AF232238 |
| dispatched homolog 2 (Drosophila) | AB051529 |
| Ras-induced senescence 1 | BF062629 |
| disabled homolog 1 (Drosophila) | NM_021080 |
| kinesin family member 26A | AK026406 |
| RAS, dexamethasone-induced 1 | AF069506 |
| cadherin, EGF LAG seven-pass G-type receptor 3 (flamingo homolog, Drosophila) | NM_001407 |
| KIAA1937 protein | AK093300 |
| transcription factor AP-2 beta (activating enhancer binding protein 2 beta) | NM_003221 |
| arachidonate 15-lipoxygenase, second type | NM_001141 |
| Guanylate binding protein 5 | BG545653 |
| insulin-like growth factor binding protein 3 | BF340228 |
| SRY (sex determining region Y)-box 30 | NM_007017 |
| chemokine (C-X-C motif) ligand 3 | NM_002090 |
| ras homolog gene family, member B | AI263909 |
| RASD family, member 2 | AF279143 |
| collagen, type II, alpha 1 (primary osteoarthritis, spondyloepiphyseal dysplasia, congenital) | X16468 |
| myosin, light polypeptide kinase /// myosin, light polypeptide kinase | NM_005965 |
| collagen, type V, alpha 3 | NM_015719 |
| Suppression of tumorigenicity 7 like | BF961733 |
| Exostoses (multiple) 1 | BC017944 |
| intercellular adhesion molecule 1 (CD54), human rhinovirus receptor | AI608725 |
| nanos homolog 1 (Drosophila) | AW970089 |
| crystallin, alpha B | AF007162 |
| peptidylprolyl isomerase (cyclophilin)-like 2 | NM_014337 |
| DEAD (Asp-Glu-Ala-Asp) box polypeptide 43 | NM_018665 |
| interleukin 20 | AF224266 |
| Kell blood group precursor (McLeod phenotype) | NM_021083 |
| DNA (cytosine-5-)-methyltransferase 3 beta | NM_006892 |
| protocadherin 10 | AI640307 |
| Similar to hypothetical protein FLJ20296 | AK098125 |
| potassium channel, subfamily K, member 15 | NM_022358 |
| endothelin 1 | BC036851 |
| CDNA FLJ11397 fis, clone HEMBA1000622 | AU144005 |
| relaxin 3 | AB076563 |
| neuronal pentraxin II | U26662 |
| TSC22 domain family 2 | AF201291 |
| neurexophilin 4 | AI933199 |
| v-maf musculoaponeurotic fibrosarcoma oncogene homolog B (avian) | NM_005461 |
| Transmembrane protein 41B | N64760 |
| transglutaminase 2 (C polypeptide, protein-glutamine-gamma-glutamyltransferase) | AL031651 |
| SMAD, mothers against DPP homolog 6 (Drosophila) | NM_005585 |
| pentraxin-related gene, rapidly induced by IL-1 beta | NM_002852 |
| Multiple C2-domains with two transmembrane regions 1 | BG250585 |
| Full open reading frame cDNA clone RZPDo834C0824D for gene HIST2H4, histone 2, H4; complete cds, incl. stopcodon | AI828075 |
| dual specificity phosphatase 9 | NM_001395 |
| dipeptidylpeptidase 4 (CD26, adenosine deaminase complexing protein 2) | M74777 |
| doublesex and mab-3 related transcription factor 1 | NM_021951 |
| hypothetical protein FLJ14351 | NM_024732 |
| forkhead box protein O6 | AI341823 |
| chromosome 9 open reading frame 140 | AW250904 |
| cytochrome c oxidase subunit 8C | AW269746 |
| cytochrome P450, family 24, subfamily A, polypeptide 1 | NM_000782 |
| family with sequence similarity 20, member A | AW291369 |
| lysosomal-associated membrane protein 3 | NM_014398 |
| HORMA domain containing 1 | AL136755 |
| synaptophysin | U93305 |
| ATP-binding cassette, sub-family A (ABC1), member 4 | U88667 |
| ADAMTS-like 2 | NM_014694 |
| histone 1, H2bd | AL353759 |
