# Supplementary material for: MAGEB2 is Activated by Promoter Demethylation in Head and Neck Squamous Cell Carcinoma
Source: PLoS One. 2012 Sep 24;7(9):e45534. doi: 10.1371/journal.pone.0045534 (PMC3454438; doi:10.1371/journal.pone.0045534)
Supplement: Table S6 — QUMSP unmethylation levels (gene of interest/bactin ×100) for 4 tested genes in primary HNSCC tumor tissues and in normal mucosa (UPPP). (DOCX) [file pone.0045534.s010.docx]

**Supplementary Table 6- QUMSP unmethylation levels for 4 tested genes in primary HNSCC tumor tissues and in normal mucosa (UPPP)**

|  | DEAD | KBGP | MAGEB2 | PPP1R14A |
| --- | --- | --- | --- | --- |
| Primary HNSCC Tumor Tissue | 473.9364 | 97.66233 | 180.445 | 45121.85 |
|  | 647.9005 | 73.2106 | 0 | 0 |
|  | 480.3968 | 89.64057 | 0 | 15192.69 |
|  | 459.5646 | 53.25613 | 2.911087 | 406878 |
|  | 425.1284 | 95.85231 | 0 | 0 |
|  | 378.9857 | 81.10194 | 0 | 24200.71 |
|  | 842.8119 | 66.84316 | 32.8983 | 5414.591 |
|  | 352.2494 | 60.66377 | 156.0679 | 2148.137 |
|  | 935.7054 | 63.56271 | 0 | 0 |
|  | 258.7856 | 39.14668 | 73.92107 | 21175.36 |
|  | 482.7957 | 55.61321 | 25.61822 | 8424.345 |
|  | 398.3993 | 55.85564 | 1.507373 | 19761 |
|  | 239.4986 | 60.8389 | 7.758434 | 24208.23 |
|  | 672.6294 | 41.91408 | 0.902619 | 0 |
|  | 232.1543 | 53.71351 | 15.3703 | 24478.45 |
|  | 1108.472 | 164.895 | 8.998934 | 221082.1 |
|  | 476.2473 | 46.48916 | 11.75474 | 82891.34 |
|  | 468.1469 | 64.25818 | 0.209371 | 4968.424 |
|  | 811.7481 | 67.36521 | 8.804634 | 1.6E+08 |
|  | 1903.74 | 177.626 | 81.32485 | 0 |
|  | 367.5412 | 40.27515 | 1.659546 | 300917.5 |
|  | 816.1312 | 38.29128 | 0 | 7.38E+08 |
|  | 637.863 | 41.85932 | 52.55882 | 8.01E+09 |
|  | 556.2474 | 78.86982 | 6.639879 | 7762.241 |
|  | 160.8586 | 56.47836 | 55.9876 | 335112.8 |
|  | 129.3951 | 29.71947 | 36.11024 | 488.1763 |
|  | 247.8672 | 52.24126 | 23.88567 | 5715.28 |
|  | 430.0362 | 58.57949 | 12.00171 | 6123.061 |
|  | 532.0347 | 62.55795 | 16.71239 | 60176.39 |
|  | 390.8415 | 46.48416 | 12.5218 | 0 |
|  | 262.8577 | 74.75213 | 27.14864 | 1408.883 |
|  | 513.4786 | 41.79929 | 7.70713 | 100394.1 |
|  | 647.5793 | 55.84031 | 1.14296 | 302573.6 |
|  | 834.4742 | 59.0584 | 2.705766 | 1937.873 |
|  | 943.179 | 76.45415 | 61.92035 | 12161.2 |
|  | 225.5005 | 41.81804 | 149.1183 | 0 |
|  | 529.2938 | 49.68911 | 133.0103 | 66885.6 |
|  | 494.8935 | 60.00392 | 32.63624 | 0 |
|  | 816.4746 | 52.92426 | 0.267455 | 142402.8 |
|  | 555.9742 | 63.58823 | 0 | 275424.3 |
|  | 692.9944 | 68.58963 | 74.6027 | 66676.19 |
|  | 1371.018 | 48.28925 | 4.158289 | 0 |
|  | 30.0467 | 31.99889 | 10.33157 | 5.1E+09 |
|  | 413.0815 | 37.67502 | 14.23102 | 1.39E+08 |
|  | 768.2104 | 46.04271 | 60.60928 | 0 |
|  | 2425.717 | 98.76734 | 6.557539 | 0 |
|  | 84.05987 | 18.15213 | 7.653575 | 732319.8 |
|  | 200.1181 | 34.85446 | 11.12436 | 21283.47 |
|  | 634.6405 | 49.78921 | 8.668881 | 7958.329 |
|  | 48.54571 | 51.24524 | 0.800023 | 2078889 |
|  | 202.5249 | 54.674 | 16.05012 | 10.80233 |
|  | 227.6864 | 46.20376 | 2.945797 | 11589.44 |
|  | 125.5278 | 51.4133 | 0 | 0 |
|  | 317.1371 | 81.35493 | 5.159015 | 0 |
|  | 271.403 | 69.89387 | 28.89444 | 11.74282 |
|  | 124.3675 | 57.72394 | 43.0786 | 0 |
|  | 42.25732 | 44.62879 | 3.824206 | 1668.676 |
|  | 425.5214 | 35.53047 | 3.746665 | 27.99389 |
|  | 397.9683 | 47.2387 | 82.62156 | 0 |
|  | 162.7607 | 39.35528 | 146.0824 | 323577.5 |
|  | 408.6156 | 49.77669 | 0.225289 | 109908.3 |
|  | 180.3941 | 39.79443 | 2.008975 | 6.61E+08 |
|  | 284.8076 | 37.53017 | 2.971423 | 45889.28 |
|  | 278.7207 | 26.1375 | 56.79211 | 40194.59 |
|  | 758.1174 | 83.2391 | 1.369951 | 1.95E+09 |
|  | 249.987 | 41.91755 | 3.975314 | 28604.86 |
|  | 775.7788 | 61.37859 | 179.6585 | 1.88E+09 |
|  | 185.2281 | 62.21575 | 5.15989 | 22070.82 |
|  | 797.7884 | 55.37859 | 0.47644 | 0 |
|  | 82.79609 | 46.5913 | 2.529767 | 1.33E+10 |
|  | 244.6351 | 59.37771 | 105.546 | 1.21E+09 |
|  | 490.0845 | 39.88366 | 7.823128 | 3120.81 |
|  | 1187.447 | 45.27891 | 3.699258 | 304.6172 |
|  | 419.4709 | 46.9694 | 0.658506 | 10183.47 |
|  | 446.104 | 70.8731 | 8.599258 | 0.737173 |
|  | 49.88606 | 25.76649 | 0.63806 | 4855320 |
|  | . | 31.33638 | 37.18482 | 1.68E+08 |
|  | . | 24.80375 | 7.07186 | . |

|  | DEAD | KBGP | MAGEB2 | PPP1R14A |
| --- | --- | --- | --- | --- |
| Normal Mucosa | 460.5504 | 72.12893 | 6.046156 | 29868.72 |
|  | 547.8093 | 48.98052 | 6.525054 | 1639438 |
|  | 708.1513 | 101.1232 | 1.206619 | 0 |
|  | 584.6945 | 70.8673 | 0.430852 | 0 |
|  | 689.4145 | 57.13741 | 3.785339 | 7721810 |
|  | 1285.239 | 46.53906 | 1.537743 | 908.1078 |
|  | 391.5455 | 68.17525 | 7.281587 | 11718.02 |
|  | 608.014 | 42.80964 | . | 19621.12 |
|  | 256.6517 | 51.70342 | 20.34573 | 0 |
|  | 1069.375 | 44.73708 | . | 0 |
|  | 511.2813 | 53.49606 | 1.285843 | 0 |
|  | 462.9199 | 41.5169 | 6.711362 | 10791.73 |
|  | 444.8209 | 53.56215 | 6.088155 | 1430.089 |
|  | 15.64559 | 77.76439 | 17.10823 | 37500.86 |
|  | 459.2642 | 54.09361 | 0.416892 | 24469.85 |
|  | 320.6798 | 50.39459 | 7.8422 | 1137794 |
|  | 345.0572 | 55.48061 | 0.471712 | 25290.1 |
|  | 1260.97 | . | 5.079806 | . |
|  | 408.5611 | . | 1.731564 | . |
