# Supplementary material for: MAGEB2 is Activated by Promoter Demethylation in Head and Neck Squamous Cell Carcinoma
Source: PLoS One. 2012 Sep 24;7(9):e45534. doi: 10.1371/journal.pone.0045534 (PMC3454438; doi:10.1371/journal.pone.0045534)
Supplement: Table S7 — Normalized RT PCR expression levels of MAGEB2 on primary HNSCC tumor tissues and normal mucosa tissues. (DOCX) [file pone.0045534.s011.docx]

**Supplementary Table 7 – Normalized RT PCR expression levels of MAGEB2 on primary HNSCC tumor tissues and normal mucosa tissues**

|  | RT PCR MAGEB2 |
| --- | --- |
| Primary HNSCC Tumor Tissue | 0 |
|  | 0.007546 |
|  | 0 |
|  | 0 |
|  | 57.63514 |
|  | 0 |
|  | 0 |
|  | 0 |
|  | 0.009698 |
|  | 0 |
|  | 0 |
|  | 63.36083 |
|  | 0.286488 |
|  | 0.008864 |
|  | 0.022665 |
|  | 0.082689 |
|  | 0.173281 |
|  | 0 |
|  | 0 |
|  | 0.567762 |
|  | 4.722393 |
|  | 0 |
|  | 0.728144 |
|  | 0.043875 |
|  | 0 |
|  | 10.71786 |
|  | 0 |
|  | 0 |
|  | 0 |
|  | 0 |
|  | 0 |
|  | 0.147173 |
|  | 0 |
|  | 0 |
|  | 0.165576 |
|  | 0 |
|  | 0 |
|  | 0 |
|  | 99.70878 |
|  | 0 |
|  | 0 |
|  | 0 |
|  | 0 |
|  | 0 |
|  | 0 |
|  | 0 |
|  | 109.1665 |
|  | 0 |
|  | 132.7851 |
|  | 0 |
|  | 131.5135 |
|  | 0 |
|  | 89.12458 |
|  | 116.9329 |
|  | 109.7173 |
|  | 97.93339 |
|  | 101.8432 |
|  | 0 |
|  | 0 |
|  | 97.55659 |
|  | 0 |
|  | 89.53514 |
|  | 0 |
|  | 0 |
|  | 0 |
|  | 0 |
|  | 0 |
|  | 99.55434 |
|  | 137.3177 |
|  | 96.04427 |
|  | 104.5259 |
|  | 99.42815 |
|  | 0 |

|  | RT PCR MAGEB2 |
| --- | --- |
| Normal Mucosa | 0 |
|  | 0 |
|  | 0 |
|  | 0 |
|  | 0 |
|  | 0 |
|  | 0 |
|  | 0 |
|  | 0 |
|  | 0 |
|  | 0 |
|  | 0 |
|  | 0.046854 |
|  | 0.294065 |
|  | 0 |
|  | 0 |
|  | 0 |
|  | 0 |
|  | 0 |
|  | 0 |
|  | 0 |
|  | 148.8521 |
|  | 92.58147 |
|  | 152.9094 |
|  | 0 |
|  | 0 |
|  | 0 |
|  | 102.2507 |
|  | 0 |
|  | 0 |
|  | 0 |
